# Supplementary material for: Phenotypic AI-based design of cell-specific small molecule cytotoxics
Source: Commun Chem. 2026 May 20;9:275. doi: 10.1038/s42004-026-02071-x (PMC13457561; doi:10.1038/s42004-026-02071-x)
Supplement: Supplementary file 1 [file 42004_2026_2071_MOESM1_ESM.pdf]

nature portfolio

Peer Review File

Phenotypic AI-based design of cell-specific small molecule cytotoxics

Corresponding Author: Professor Patrick Aloy

This file contains all reviewer reports in order by version, followed by all author rebuttals in order by version.

Version 0:

Reviewer comments:

Reviewer #1

(Remarks to the Author)
The authors present a study combining high-throughput screening with predictive and generative modeling to identify novel chemical matter with cell-specific inhibitory activity. Concretely, the authors initially perform a high-throughput screen informed by chemical probes from literature to find initial inhibitors across a panel of pancreatic cancer cell lines. With these data, they built predictive machine learning models based on bioactivity profiles that are subsequently used to steer generative chemistry models that design novel cell-specific inhibitors. The novel molecular designs are experimentally tested by proxy through accessible analogs. The results show that their predictive and generative pipelines are able to discover cell-specific novel inhibitors.

This is a very comprehensive study illustrating the potential of phenotypic data as a guiding signal for the design of novel inhibitors. The results with their in silico bioactivity profiles reinforce prior results (Martin et al., J. Chem. Inf. Model. 2019, 59, 10, 4450–4459; Godinez et al. Nat Mach Intell, 2022, 4, 180–186) indicating these bioactivity profiles to be effective at both identifying potential inhibitors in existing chemical libraries as well as steering the design of novel molecules.

One main issue is the lack of a breakdown of the performance of the de novo design approach with respect to the different specificity scenarios/exercises in the main text. While the number of analogs of novel molecules is low, it would still be informative to know whether certain specificity scenarios were more amenable to the de novo approach, or whether most successful compounds primarily emerged in, for example, the HEK293 inhibition scenario. Relatedly, it would be useful to understand how many compounds in the initial HTS set actually matched the multi-(or dual-)objective specificity requirements of each exercise, and whether that number correlates with the observed hit rates.

Another minor issue pertains to the comparison of cell profiles relying on a Euclidean distance that is non-normalized as opposed to using a cosine distance that is inherently normalized and would also remain numerically closer to the Jaccard index ra nge. Along similar lines, binarizing the cellular inhibition profiles and computing equivalent Jaccard index would strengthen the interpretation of these distance comparisons.

Reviewer #2

(Remarks to the Author)
In their work “Phenotypic AI-based design of cell-specific small molecule cytotoxics” the authors use AI-based approaches to explore the chemical space for optimal cytotoxics. Based on in-house generated phenotypic screening data, they train machine-learning classifiers which are then used as scoring functions in a generative, reinforcement learning-based framework, REINVENT. Machine learning classifier training seems successful based on the shown metrics and the whole generative framework outputs molecules that show in many of the cases 10-fold or more specificity in killing target cells. I fully agree AI-based tools are needed to explore the chemical space after phenotypic screening, and this manuscript is a good example of such an approach. Please see my concerns for this work listed below:

Major:
- Currently it remains vague how specific the hit compounds actually are. Computational models are only able to learn drug-cell line specificities if a signal for this exists in the training dataset. The HTS hits in Figure1 should be better described and

a more transparent view into this data should be provided. Apparently the screen was able to discover PC specific inhibitors (392 hits) but how efficient these are at killing PC cells vs control is not really shown/plotted. Could the authors show examples of the PC specific molecules starting by plotting their efficacy (waterfall plot: Y-axis cell killing, X-axis molecules) and try to explore why these molecules are specific to PC? What were the most specific molecules?

-Fig1e: These correlations are based on very few cell lines so I would not suggest making strong assumptions based on them. Rather, I would suggest the authors use this space in Figure 1 to more transparently discuss the hits and their specificities.

-Fig2: As far as I understand, the GLOBAL representation of molecules is inferred using AI. Does this risk data leakage or artificially boosting training scores of the models? Because both measured and inferred features are used, the authors should show feature importance ranking and try to assess which types of features seem most predictive.

-Fig. 2c: In addition to higher hit enrichments, do you find more potent molecules or molecules with new scaffolding? Currently this is not being shown.
How did the authors prevent reinforcement learning moving outside the chemical domain where the scoring function is applicable?

-When calculating IC50 values (Fig5), the concentrations used for many of the molecules do not achieve 50% inhibition making curve fitting difficult. In addition, 100% inhibition is not achieved with most of the titration series. The authors should extend the used concentrations far enough for reliable curve fitting and IC50 estimation.

-Although testing close analogues of the predicted molecules can be OK, in many of the cases the Tanimoto-similarities are not that great and this part does not really validate the predictions in my opinion. This leaves a feeling that the project is merely a computational exercise. To get past this, could the authors test 1 or few of the predicted molecules across all PC lines to compare if they have more selectivity than the close analogues? In some of the cases it might be possible to achieve nanomolar inhibition while being specific.

Minor:
-What are "default structural alerts" and how was this implemented?

-"After the cross-validation, we used the complete set of positives as training, without a validation split, to train a final model for each cell line using CC bioactivity descriptors, as they showed superior performance." Why was this done and how was this model superiority tested?

- Odds ratio vs HTS" is not well explained. How was this metric calculated?

Version 1:

Reviewer comments:

Reviewer #2

(Remarks to the Author)
The authors have now provided more in-depth analysis into the training data and especially into the discovered hit molecules. The new results highlight the advantages of the used generative framework on phenotypic drug discovery and I will suggest this work to be published without further delay. Thank you for the authors for their hard work producing this interesting article.

Open Access This Peer Review File is licensed under a Creative Commons Attribution 4.0 International License, which permits use, sharing, adaptation, distribution and reproduction in any medium or format, as long as you give appropriate credit to the original author(s) and the source, provide a link to the Creative Commons license, and indicate if changes were made.
In cases where reviewers are anonymous, credit should be given to 'Anonymous Referee' and the source.
The images or other third party material in this Peer Review File are included in the article's Creative Commons license, unless indicated otherwise in a credit line to the material. If material is not included in the article's Creative Commons license and your intended use is not permitted by statutory regulation or exceeds the permitted use, you will need to obtain permission directly from the copyright holder.
To view a copy of this license, visit https://creativecommons.org/licenses/by/4.0/

Point-by-point responses to Reviewer's comments

COMMSCHEM-25-1089-T "Phenotypic AI-based design of cell-specific small molecule cytotoxics" by Rojas-Granado et al.

We would first like to thank the Editor and Reviewers for their critical reading of our manuscript and the constructive feedback. We have now addressed in detail all the raised issues which has, indeed, improved the robustness and clarity of our paper. In brief:

- We analyzed the cell selectivity of the hit compounds identified in the HTS, and compared it to the quality of the generative models for each task.
- We reanalyzed the relationship between cytotoxicity and GEx profiles, using Euclidean, cosine and Jaccard distances.
- We explored whether there could be data leakage between the Signaturizers and the cell-specific cytotoxicity prediction models, discarding this possibility.
- We explored the potency and chemical novelty of the identified and generated compounds.
- We clarified that, indeed, 15 of the 45 experimentally tested compounds are the very same that were generated by our methodology, and we analyzed the relationship between the chemical similarity between generated and tested compounds and their activity.
- Finally, we have clarified several issues in the revised version of the manuscript, including the calculation of Odd ratios and the structural alerts considered, among others.

Overall, we have modified Figure 1, we have included 5 new supplementary figures and 2 supplementary tables, and we have extended the discussion of the m/s to address in detail all the points raised by the Reviewers.

Please find below a point-by-point response to Reviewers' comments. Major edits are highlighted in yellow in the revised version of the manuscript.

Reviewer #1

The authors present a study combining high-throughput screening with predictive and generative modeling to identify novel chemical matter with cell-specific inhibitory activity. Concretely, the authors initially perform a high-throughput screen informed by chemical probes from literature to find initial inhibitors across a panel of pancreatic cancer cell lines. With these data, they built predictive machine learning models based on bioactivity profiles that are subsequently used to steer generative chemistry models that design novel cell-specific inhibitors. The novel molecular designs are experimentally tested by proxy through accessible analogs. The results show that their predictive and generative pipelines are able to discover cell-specific novel inhibitors.

This is a very comprehensive study illustrating the potential of phenotypic data as a guiding signal for the design of novel inhibitors. The results with their in silico bioactivity profiles reinforce prior results (Martin et al., J. Chem. Inf. Model. 2019, 59, 10, 4450–4459; Godinez et al. Nat Mach Intell, 2022, 4, 180–186) indicating these bioactivity profiles to be effective at both identifying potential inhibitors in existing chemical libraries as well as steering the design of novel molecules.

We thank the Reviewer for their positive comments, and for acknowledging the comprehensiveness and potential of our study.

1. One main issue is the lack of a breakdown of the performance of the de novo design approach with respect to the different specificity scenarios/exercises in the main text. While the number of analogs of novel molecules is low, it would still be informative to know whether certain specificity scenarios were more amenable to the de novo approach, or whether most successful compounds primarily emerged in, for example, the HEK293 inhibition scenario.

We thank the Reviewer for their comment. To evaluate whether certain specificity scenarios were more amenable to the de novo design approach, we examined the performance of each generative exercise individually, considering both predicted activity and experimentally validated outcomes. This information was already contained in Fig. 3b (top panel) and Fig. 4a, but we now explicitly describe the results of our analyses in the main text.

We observe that, based on our prediction scores, the different generative exercises display different yields in terms of the number of high-scoring generated molecules, ranging from 0.42% in HEK293 vs BXPC3 to 2.68% in BXPC3 vs CAPAN2 exercise. Additionally, the experimental. validation provides a complementary perspective: the majority of validated hits originate from the BXPC3 vs CAPAN2 exercise (12 hits), in perfect agreement with the prediction scores, followed by HEK293 vs CFPAC1 (5 hits) (Figure 4a). For three more exercises we were able to validate 1 hit (BXPC3 vs HEK293, HEK293 vs BXP3 and HEK293 vs CAPAN2), and for the remaining two we didn't succeed in the selectivity objective (CAPAN2 vs HEK293 and CAPAN2 vs CFPAC1).

To enable straightforward comparisons between the different specificity scenarios, we now include table R1, providing detailed information about results from HTS, generative models and IRB library prediction including the number of compounds generated, high-scoring, tested and hit per exercise. We substituted Supplementary Table 9 in the m/s by this one containing more comprehensive information.

Exercise	HTS confirmation		Generative models				IRB library predictions		
	Tested	Active	Unique	High scoring (% over unique)	Tested	Active	Total	High scoring – Tested	Active
BXPC3 vs CAPAN2	751	84	5,111	137 (2.68%)	16	12	133,672	7	1
BXPC3 vs HEK293	751	54	5,067	47 (0.93%)	22	1	133,672	5	1
CAPAN2 vs HEK293	751	82	18,707	100 (0.53%)	1	0	133,672	27	4
CAPAN2 vs CFPAC1	751	84	9,235	121 (1.31%)	1	0	133,672	17	4
HEK293 vs BXPC3	751	78	8,943	38 (0.42%)	1	1	133,672	3	1

HEK293 vs CAPAN2	751	100	4,603	40 (0.87%)	1	1	133,672	2	1
HEK293 vs CFPAC1	751	79	8,692	60 (0.69%)	11	5	133,672	4	0

Table R1. Molecule counts for all selectivity exercises in each part of the project.

2. Relatedly, it would be useful to understand how many compounds in the initial HTS set actually matched the multi- (or dual-) objective specificity requirements of each exercise, and whether that number correlates with the observed hit rates.

We thank the Reviewer for this relevant comment. To investigate the relationship between HTS outcomes and generative success, Table R1 also shows a breakdown of the number of hits in HTS but also in the different phases of the generation approach. Moreover, we have plotted the results in Figure R1 to show the number of molecules meeting selectivity criteria for each cell-line pair and their correlation with generative performance.

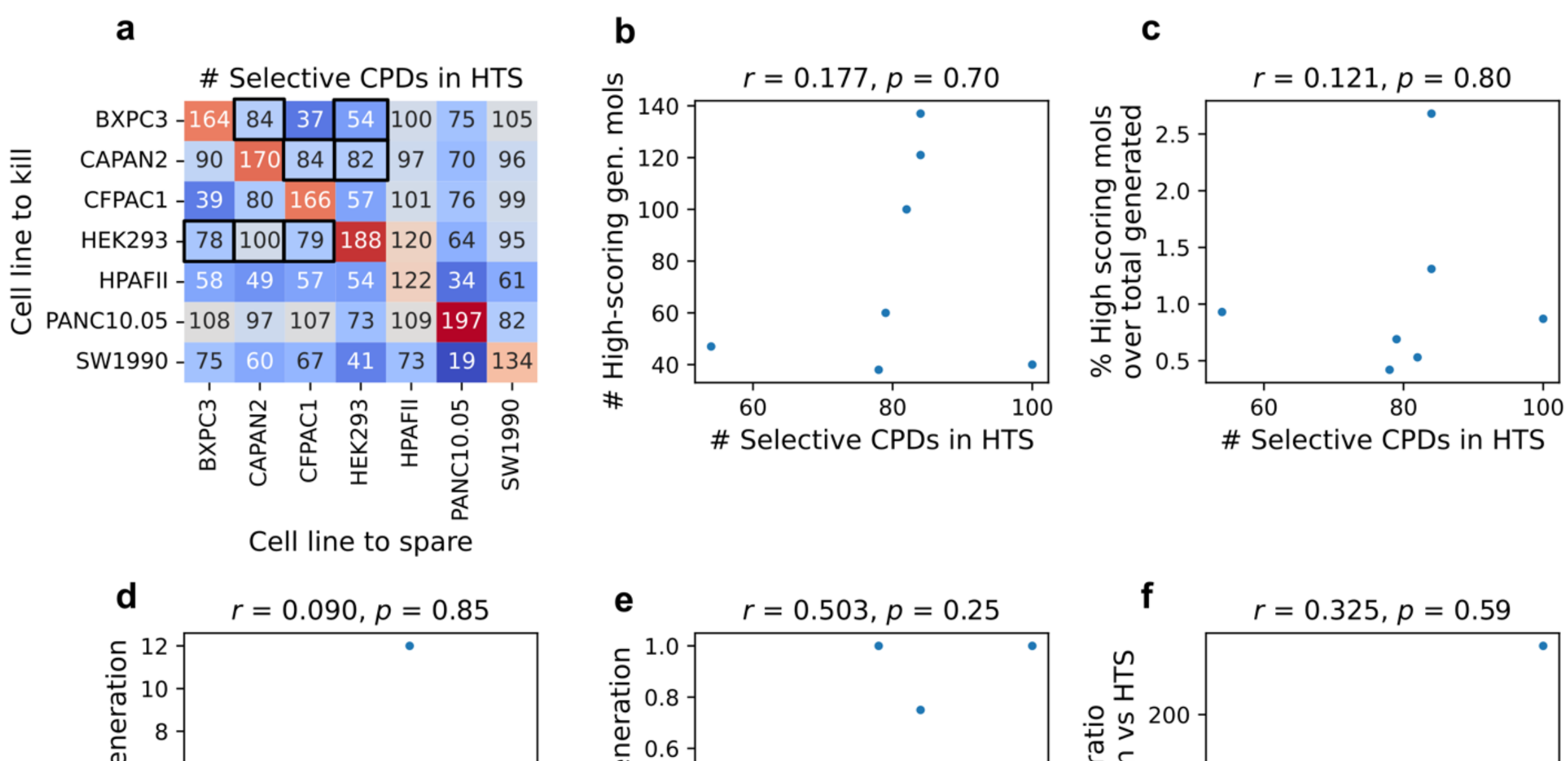

Figure R1. Correlation between the number of dual-hits in the HTS and the performance of the prediction models. (a) Number of molecules in the HTS confirmation assay (i.e., training data, 751 compounds) that matched the indicated selectivity criteria for each cell line pair. The diagonal shows the number of active molecules for the individual cell line for reference, instead of empty values. Highlighted squares are the selected exercises. **(b-f)** show correlation between the number of actives in HTS vs: (b) number of high-scoring generated molecules; (c) % high scoring generated molecules over total molecules generated; (d) number of hits in the generative approach; (e) hit rate per generative exercise; (f) odds ratio in hit discovery rate, generative approach vs HTS.

Panel **(a)** show the number of molecules in the HTS confirmation assay (i.e. the 751 compounds used in the models´ training) that matched the indicated selectivity criteria for each cell line pair. We then explore the potential correlation between the number of actives for each specificity exercise in the HTS and the performance of the predictive models. Despite the limited number of data points, we observe no clear relationship between the number of HTS actives for each exercise and overall generative performance, including absolute hit counts, hit rates, or odds ratios. For instance: both the exercise with the highest number of actives in HTS (HEK293 vs CAPAN2, 100 actives) and the one with the lowest number (BXPC3 vs HEK293, 54 actives) yielded only one experimental hit each. On the other hand, the most successful exercises, BXPC3 vs CAPAN2 (12 hits) and HEK293 vs CFPAC1 (5 hits), had intermediate numbers of actives in the HTS, 84 and 79 actives respectively.

Overall, these results highlight that we cannot assume a linear relationship between performance in HTS and generation. Considering that the cytotoxicity models used for steering the generation process were trained on the individual cell lines before being combined into a dual-objective approach, the lack of correlation with the HTS data is not surprising.

Since this is, indeed, a relevant comment, we have now included the breakdown of the number of hits per selectivity exercise in each part of the project (HTS, generation and prediction on IRB library) in Supplementary Table 9, and the heatmap with the number of active molecules per exercise in the HTS as part of Figure 1.

3. Another minor issue pertains to the comparison of cell profiles relying on a Euclidean distance that is non-normalized as opposed to using a cosine distance that is inherently normalized and would also remain numerically closer to the Jaccard index range. Along similar lines, binarizing the cellular inhibition profiles and computing equivalent Jaccard index would strengthen the interpretation of these distance comparisons.

We thank the Reviewer for this comment. We agree that cosine distance is inherently normalized and facilitates comparison with metrics such as the Jaccard index. Our original choice of Euclidean distance was due to the fact that, in both gene expression and cytotoxicity data, the magnitude of the cellular response carries relevant information in addition to the overall profile direction. We have now complemented the original analysis by computing cosine and Jaccard distances (Figure R2), confirming that there is a weak correlation between cytotoxicity and GEx or mutation profiles when considering HEK293 and U2OS control cells, but the signal disappears when only pancreatic cancer cell lines are analyzed. We report the results of these analyses in Extended Data Figure 3.

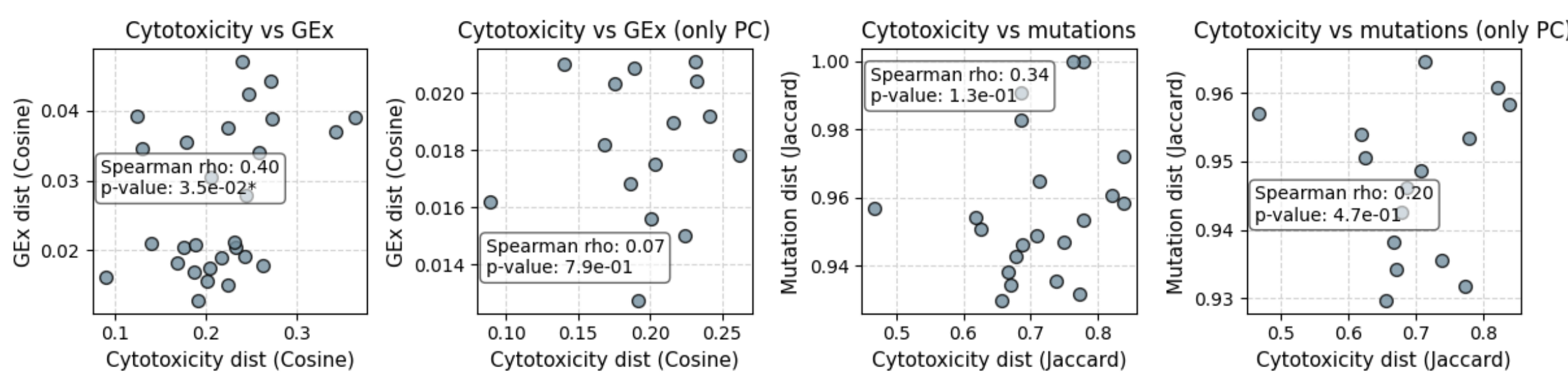

Figure R2. Correlation between cell line distances in cytotoxicity and gene expression (GEx) or mutational profiles.

Reviewer #2

In their work "Phenotypic AI-based design of cell-specific small molecule cytotoxics" the authors use AI-based approaches to explore the chemical space for optimal cytotoxics. Based on in-house generated phenotypic screening data, they train machine-learning classifiers which are then used as scoring functions in a generative, reinforcement learning-based framework, REINVENT. Machine learning classifier training seems successful based on the shown metrics and the whole generative framework outputs molecules that show in many of the cases 10-fold or more specificity in killing target cells. I fully agree AI-based tools are needed to explore the chemical space after phenotypic screening, and this manuscript is a good example of such an approach. Please see my concerns for this work listed below:

We thank the Reviewer for assessing our work and for acknowledging the relevance of our study.

Major:

1. Currently it remains vague how specific the hit compounds actually are. Computational models are only able to learn drug-cell line specificities if a signal for this exists in the training dataset. The HTS hits in Figure1 should be better described and a more transparent view into this data should be provided. Apparently, the screen was able to discover PC specific inhibitors (392 hits) but how efficient these are at killing PC cells vs control is not really shown/plotted. Could the authors show examples of the PC specific molecules starting by plotting their efficacy (waterfall plot: Y-axis cell killing, X-axis molecules) and try to explore why these molecules are specific to PC? What were the most specific molecules?

We thank the Reviewer for their comment, and we agree that the specificity of the hit compounds in the HTS was not addressed in detail in the first version of the manuscript.

However, before addressing the specificity question, we would like to highlight that the primary goal of the project was to explore the ability of generative models to design new compounds with specific cytotoxic profiles, as a theoretical exercise to validate the methodology, but not as a therapeutic approach to find molecules specifically targeting PC cells without affecting non-PC ones. Thus, the primary screening was designed to find cytotoxic molecules against a panel of 7 pancreatic cancer cell lines, and we only included the HEK293 and U2OS controls during the confirmation phase, so that we could characterize the broader activity profile of the 751 hit compounds. Please, note that our reinforcement learning approach, ideally, should be able to learn which compounds affect every cell line and combine their features to design new chemical entities with the desired cytotoxic properties, even if no molecules witch such properties are present in the training.

Additionally, we would also like to clarify that the 392 hits identified during the confirmation screening include all molecules active against at least one pancreatic cancer (PC) cell line, regardless of their activity on control lines. Of these, 19 compounds were active exclusively on the AsPC-1 cell line, removed from subsequent analysis due to cell identity issues as described

in Methods. Therefore, our final dataset contains 373 compounds active against any of the 6 remaining PC cell lines. We mistakenly reported the numbers including the AsPC-1 cell line and consequently have now updated the manuscript and methods to reflect these corrected values. As these extra 19 compounds were active exclusively on AsPC-1, none of the subsequent sections of the manuscript were affected by the change. We have now clarified all these points in the m/s, and we thank the Reviewer for their comment.

To specifically address the Reviewer´s point regarding the selectivity of hit compounds in the HTS, we found that 149 of the 373 hits were active in any of the PC cell lines without affecting the controls at the established thresholds. Figure R3a shows the cytotoxicity profile against controls of the 373 hit molecules, while Figure R3b shows the activity profile of the remaining 378 molecules that did not show cytotoxicity in PC cells. Finally, the UpSet plot in Figure R3c depicts the profile of the 149 hits that showed cytotoxicity in PC cell lines but did not affect controls. We only found 4 molecules that show cytotoxic activity across all PC lines without affecting any of the controls at the established thresholds. We have depicted their chemical structures in the UpSet plot in Figure R3c, and their % inhibition with respect to the established cytotoxicity thresholds is shown in Figure R4.

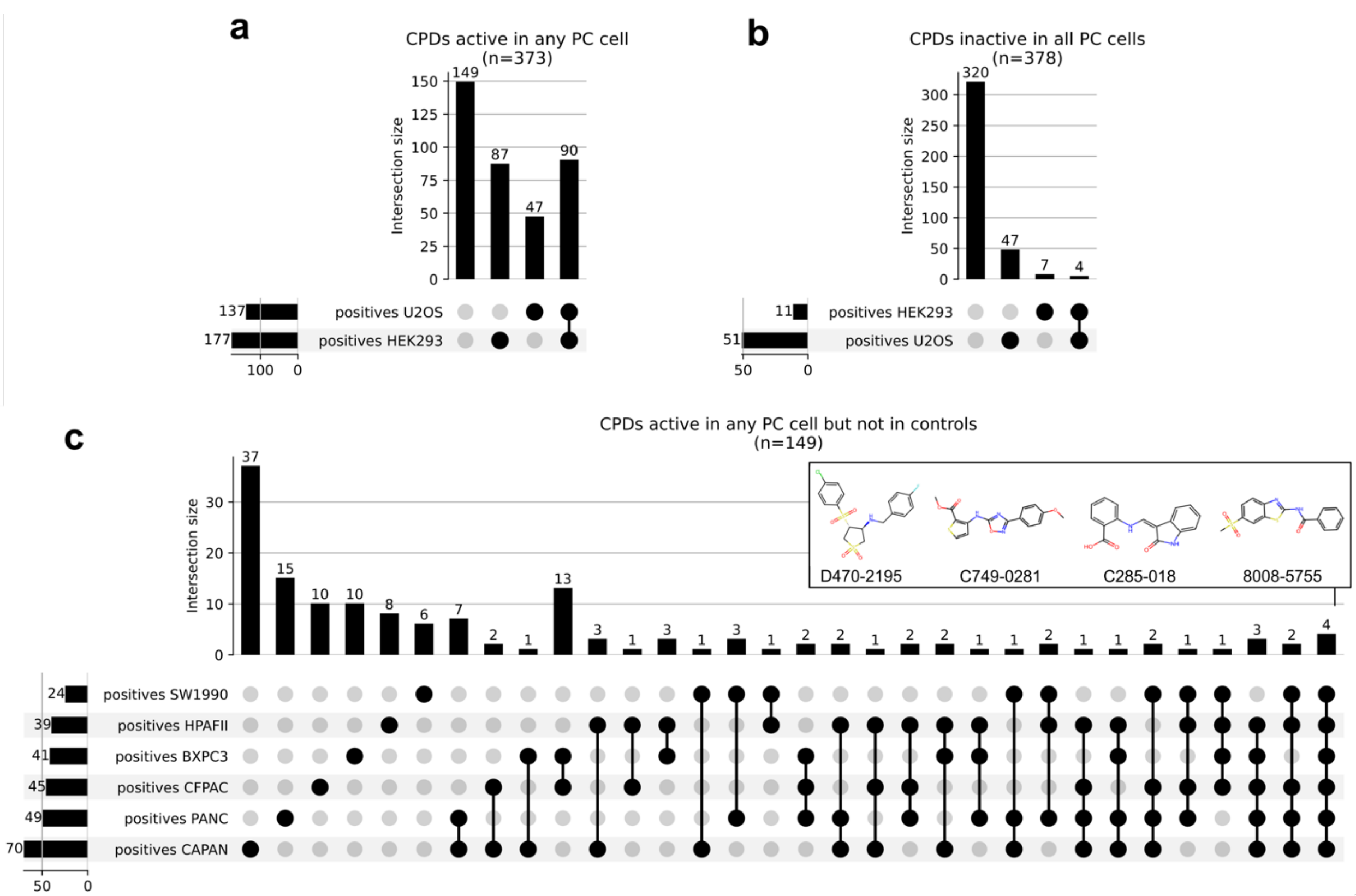

Figure R3 | Cytotoxicity profile of molecules in the HTS. (a) compounds active in any PC cell line (373). **(b)** compounds inactive in all the PC cells simultaneously (378). **(c)** compounds active in any PC cell line but not in controls (149). The displayed structures correspond to molecules that simultaneously kill all the 6 PC cell lines without affecting any of the controls.

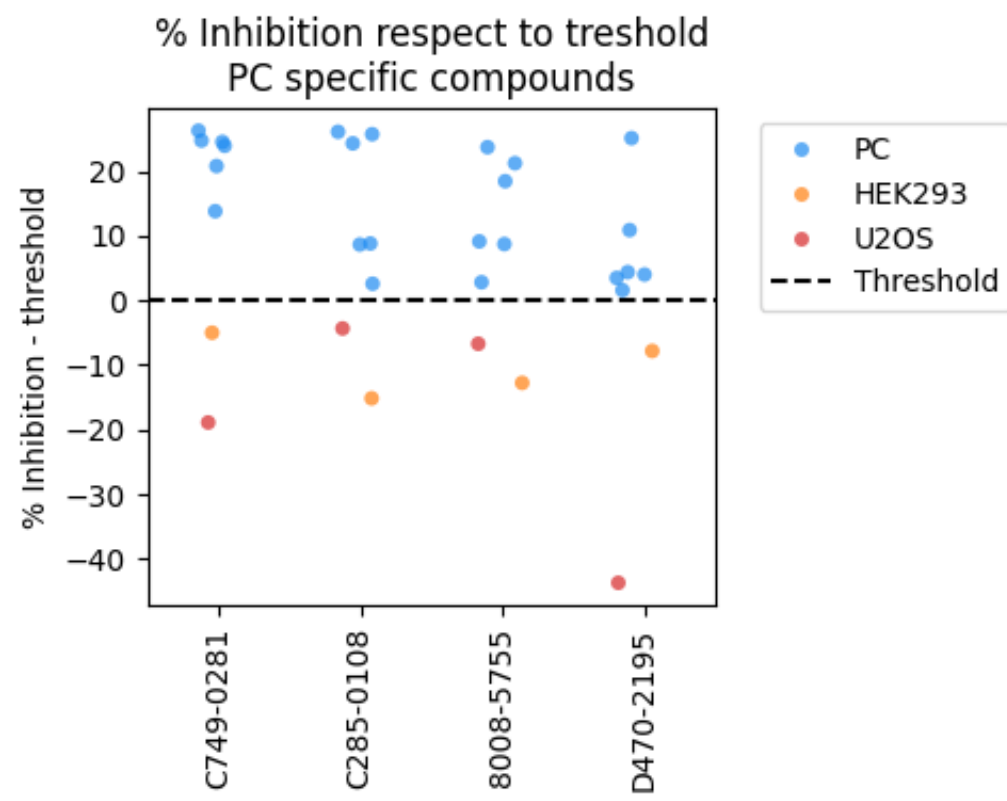

Figure R4 | Cytotoxic activity (% inhibition) with respect to thresholds for the 4 PC-specific molecules found in the HTS.

If we analyze pairwise selectivity, Figure R5 shows how many compounds are active in one cell line but not another, accounting for all the possible combinations and highlighting the selected exercises for the generative approach. The two bottom rows correspond to molecules that affect the control cell lines and not others, whereas the two last columns correspond to molecules sparing the controls.

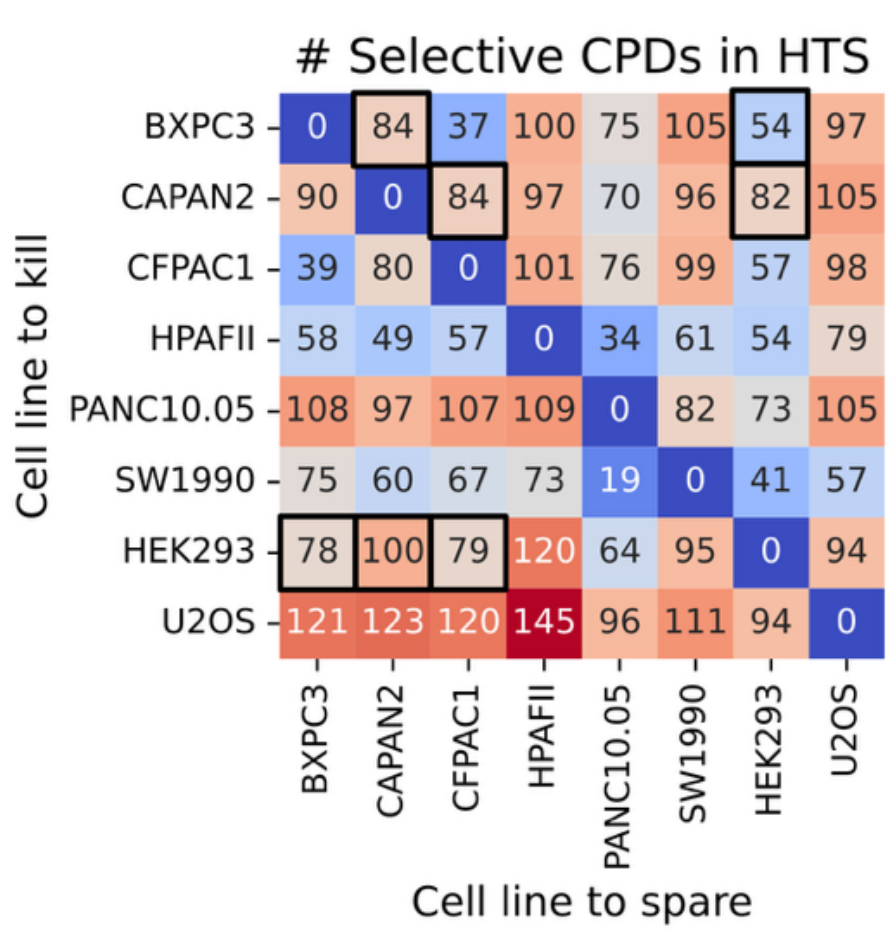

Figure R5 | Pairwise cytotoxic selectivity of compounds in the HTS.

About the efficacy of the 373 molecules active in any of the PC cell lines, Figure R6 contains the waterfall plots requested by the Reviewer, showing the %inhibition across cell lines. As depicted in Figure 1b and discussed in the main text, the control cell lines proved more sensitive to inhibition than the pancreatic cancer lines, which are overall much more resistant to treatment, and therefore the activity thresholds in those lines are set higher.

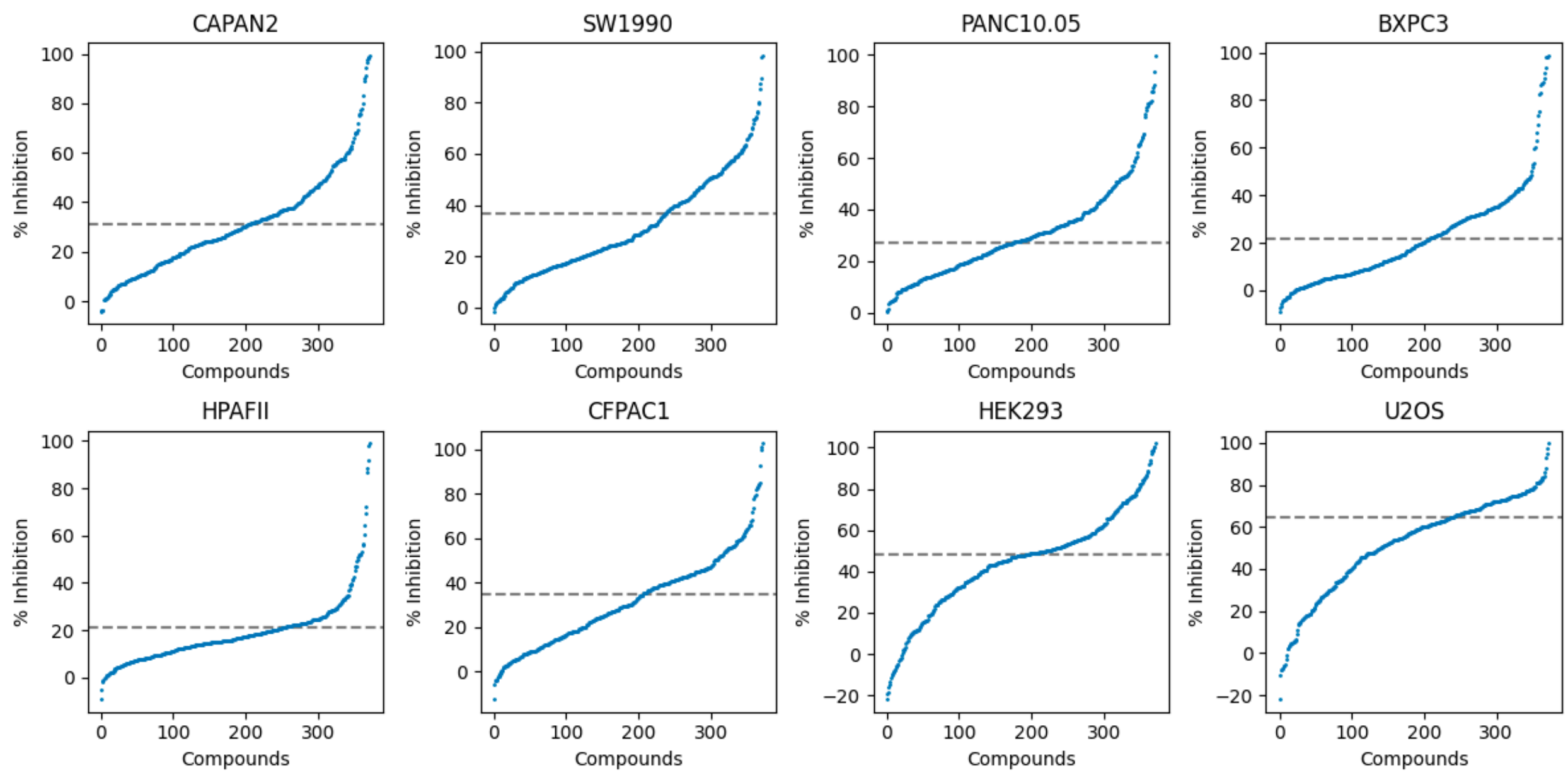

Fig. R6 | Waterfall plots showing cytotoxic activity (%inhibition) for the 373 molecules active against any PC cell line in the HTS.

In addition to this extra analysis, Figure 1f shows scaffolds associated with activity on PC cells, and Extended Data Fig 4 goes into further details on the most prevalent scaffolds.

About mechanistic insights on the selectivity effect, please note that the library comes from a discovery subset from ChemDiv so most of the molecules lack annotations or bioactivity characterization, which makes it difficult to elucidate why they target one cell line and not another.

We thank the Reviewer again for their comment and hope we have shed some light on the selectivity profile of the hits in the HTS. We have now removed the scatter plots in Figure 1e and included them as part of Extended Data Figure 3. Additionally, we have substituted Figure 1d, representing pair-wise overlap in active molecules across cell lines by the heatmap in Figure R5 representing the number of selective molecules per cell line pair, which is more relevant for the subsequent sections of the m/s.

2. Fig1e: These correlations are based on very few cell lines so I would not suggest making strong assumptions based on them. Rather, I would suggest the authors use this space in Figure 1 to more transparently discuss the hits and their specificities.

We thank the Reviewer for their comment. While the correlation analysis in Figure 1e is based on a limited number of data points, we believe these correlations remain informative for identifying potential trends across the tested panel. Following the Reviewer´s suggestion we have moved the correlation plots from the main text to the Extended Data as part of Extended Data Figure 3, and have replaced Figure 1d by the analysis on hit selectivity shown in Figure R5. We hope these changes provide a clearer view of how the hits affect the different pancreatic cancer cell lines and controls.

3. Fig2: As far as I understand, the GLOBAL representation of molecules is inferred using AI. Does this risk data leakage or artificially boosting training scores of the models? Because both measured and inferred features are used, the authors should show feature importance ranking and try to assess which types of features seem most predictive.

We thank the Reviewer's comment and understand their concern. As described in the *Methods - Compound descriptors* section, the CC_GLOBAL descriptors are generated using the Signaturizers[1], a collection of deep networks that generate bioactivity descriptors from SMILES representations. Thus, they are just a general approach to generate small molecule descriptors, and they have not been trained (or fine-tuned) in anyway with the data generated in this project. The Signaturizers are trained on the full Chemical Checker[2,3] universe, which comprises over 1,2M molecules, 18,529 of which had explicit cytotoxicity information (D2 bioactivity space). Only 2 of the 384 active compounds used to train our models (373 active in any PC line + 11 active only in HEK293) are present in the Chemical Checker D2 space. Thus, this minimal overlap (i.e. 2 out of 1,2M molecules), and the lack of specific training of the Signaturizers for this exercise, make data leakage extremely unlikely.

Additionally, and following the Reviewer´s suggestion, we studied impact of cytotocicity data in the Chemical Checker (i.e. D2 space) in the individual cytotoxicity models built. As described in *Methods – Compound descriptors* and *Methods - Cell-line sensitivity prediction*, the original Signaturizer inferred descriptors have 128-dimensions per bioactivity space, i.e., 3,200 accounting for the 25 bioactivity spaces. To train our models, we first used a k-best selector from scikit-learn to extract the 128 more relevant features out of the initial 3,200. From these 128, the number of features coming from the D2 space ranges from 0 to 19 depending on the cell line (Figure R7).

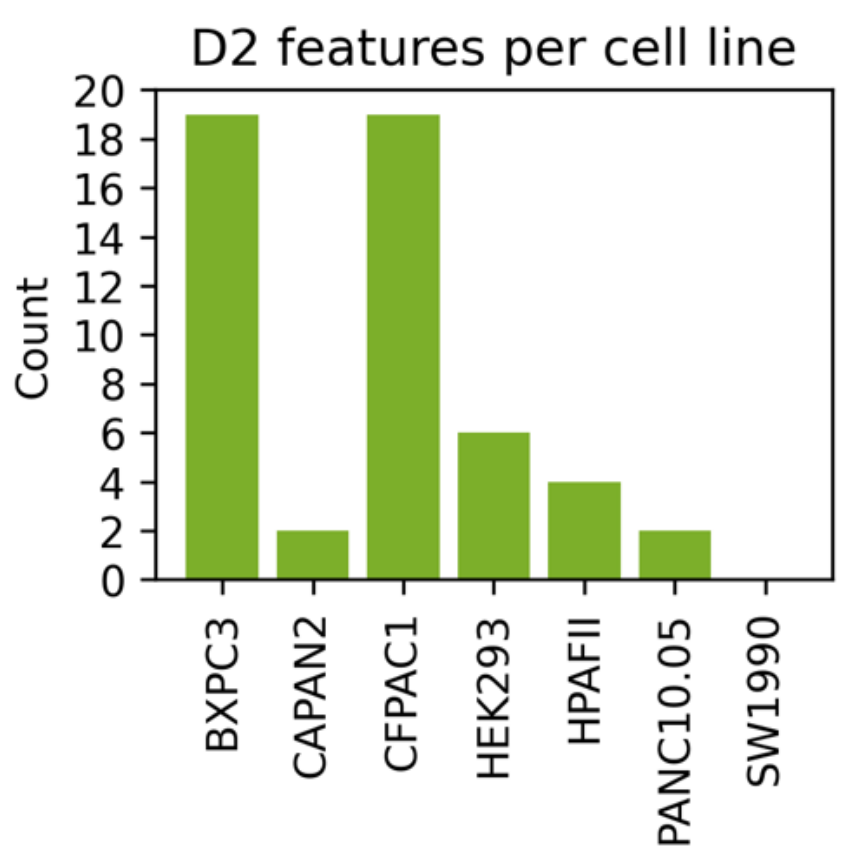

Figure R7 | Number of features coming from the D2 space per cell line (i.e., per prediction model).

Moreover, we have now analyzed the contribution of each Chemical Checker bioactivity space to each ExtraTrees classifier to predict binary cell cytotoxicity trained on the available HTS dataset (Figure R8). We observe that most informative features come from chemistry-related spaces (A)

complemented by other bioactivity spaces, especially those related to targets (B5), networks (C1,2) and cell-based assays (D1,2,4).

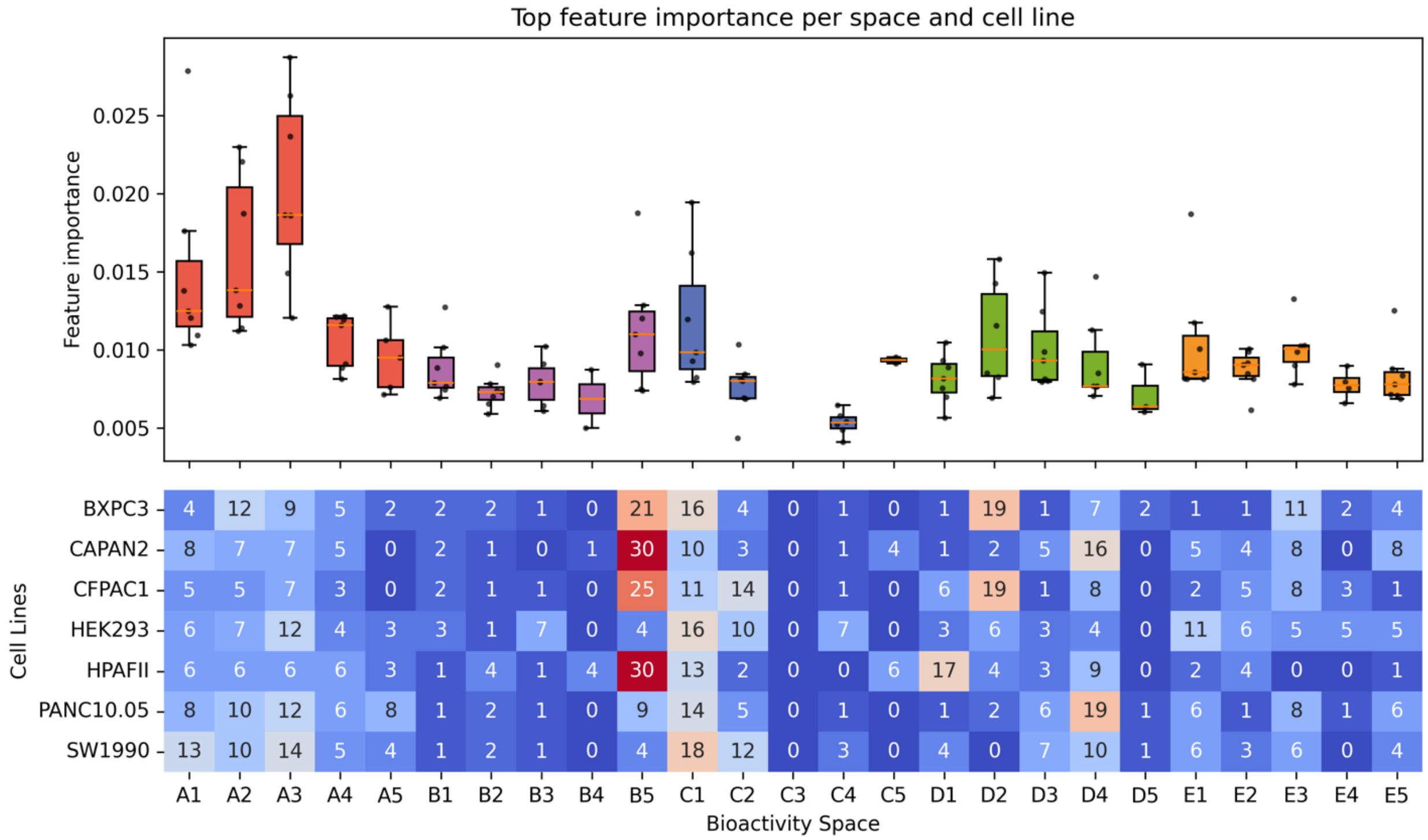

Figure R8 | Summary of feature importance per prediction model. The top panel displays the maximum feature importance per cell line (prediction model) across the CC bioactivity spaces; specifically, each dot represents the highest importance value assigned to any feature within that space for a given model. The bottom panel shows the number of features coming from each bioactivity space per cell line. Further details on the original data in each bioactivity space and how the final Signaturizer features were obtained is available in the Chemical Checker and related publications[1–3].

With these, we hope we have addressed the Reviewer's concern about data leakage and thank them for their feedback. We now stress in the Methods the inferred nature of the Signaturizers descriptors and the lack of overlap between the CC chemical space (where the Signaturizers were trained on) and the molecules in our dataset.

4. Fig. 2c: In addition to higher hit enrichments, do you find more potent molecules or molecules with new scaffolding? Currently this is not being shown.

We thank the Reviewer for their question. Please, note that our predictors were solely trained on binary activity labels and not quantitative activity measures (i.e. active/inactive at 10uM). Thus, the models are not explicitly designed to preferentially identify more potent molecules, but rather to enrich for active compounds at the given thresholds. Furthermore, as we have only measured activity at a single concentration (i.e. 10uM), we cannot directly evaluate potency, and we can only use as proxy the % inhibition at this particular concentration.

To address the Reviewer's point, we have now explicitly evaluated whether the identified hits include molecules with comparable or higher % activity than training actives (Figure R9). We find that the % inhibition of the compounds in the validation set is comparable to that of the HTS actives, including molecules reaching >98% inhibition both in the HTS and the validation.

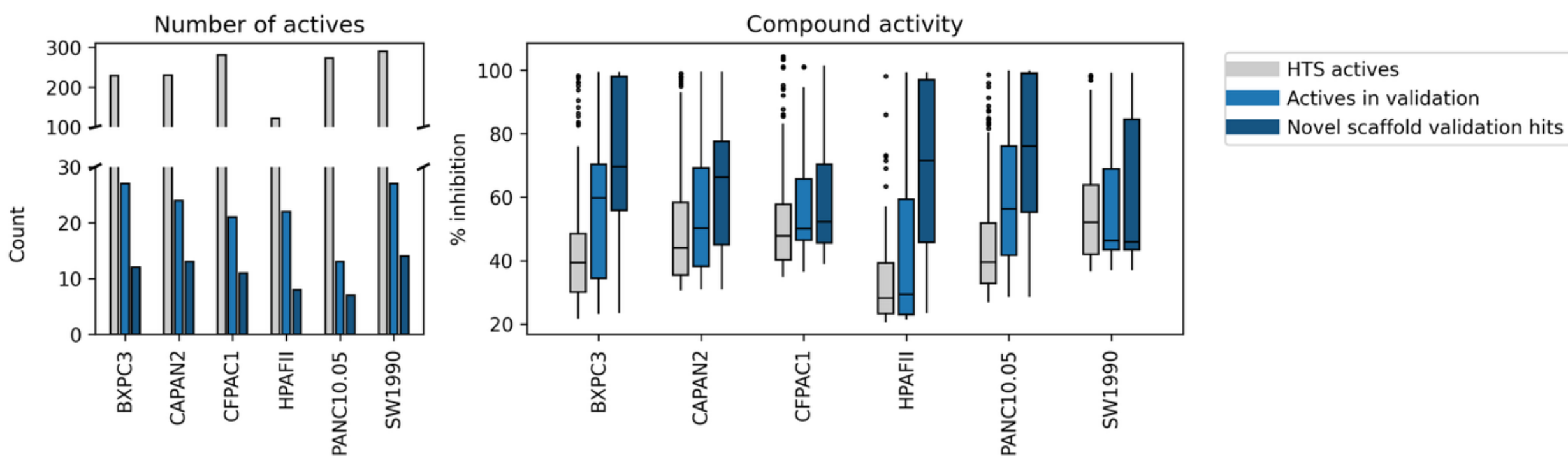

Figure R9 | Comparison of the inhibitory potential of HTS and validation hit compounds. The left panel shows the number of active molecules identified in both screenings, alongside the number of validated hits possessing novel scaffolds in comparison to the original HTS actives. The right panel shows the corresponding cytotoxic activity (%inhibition) for these molecules.

Regarding structural novelty of the hits in the validation exercise, when looking at the annotated scaffold groups in the library we identified 20 molecules representing 12 new scaffold groups not previously detected among the HTS actives. Additionally, we found 26 molecules bearing 14 scaffolds common to the actives from both screenings. These 12 new scaffolds and 14 known ones are depicted below in Figures R10 and R11, respectively.

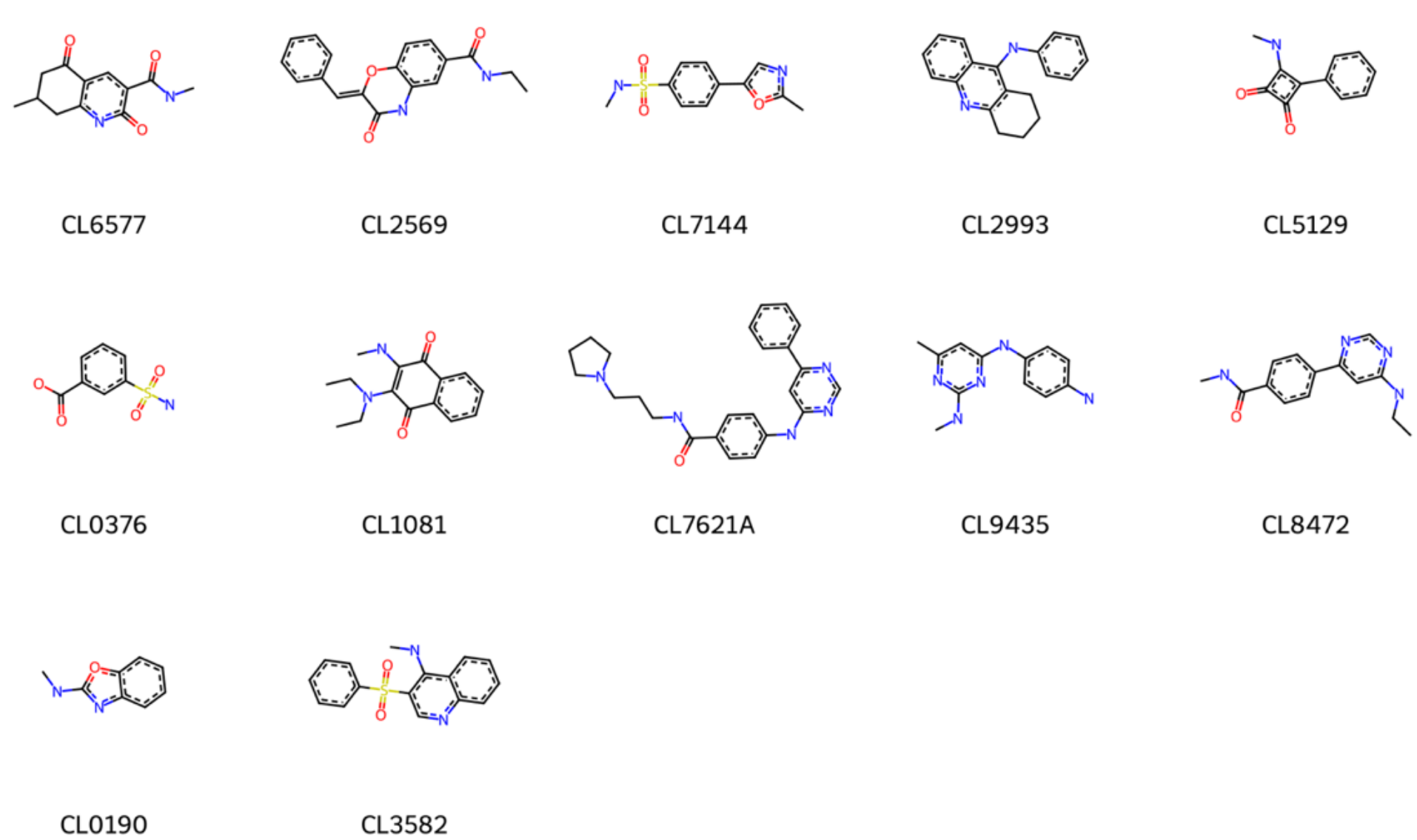

Figure R10 | New scaffold groups in active molecules identified during validation of prediction models.

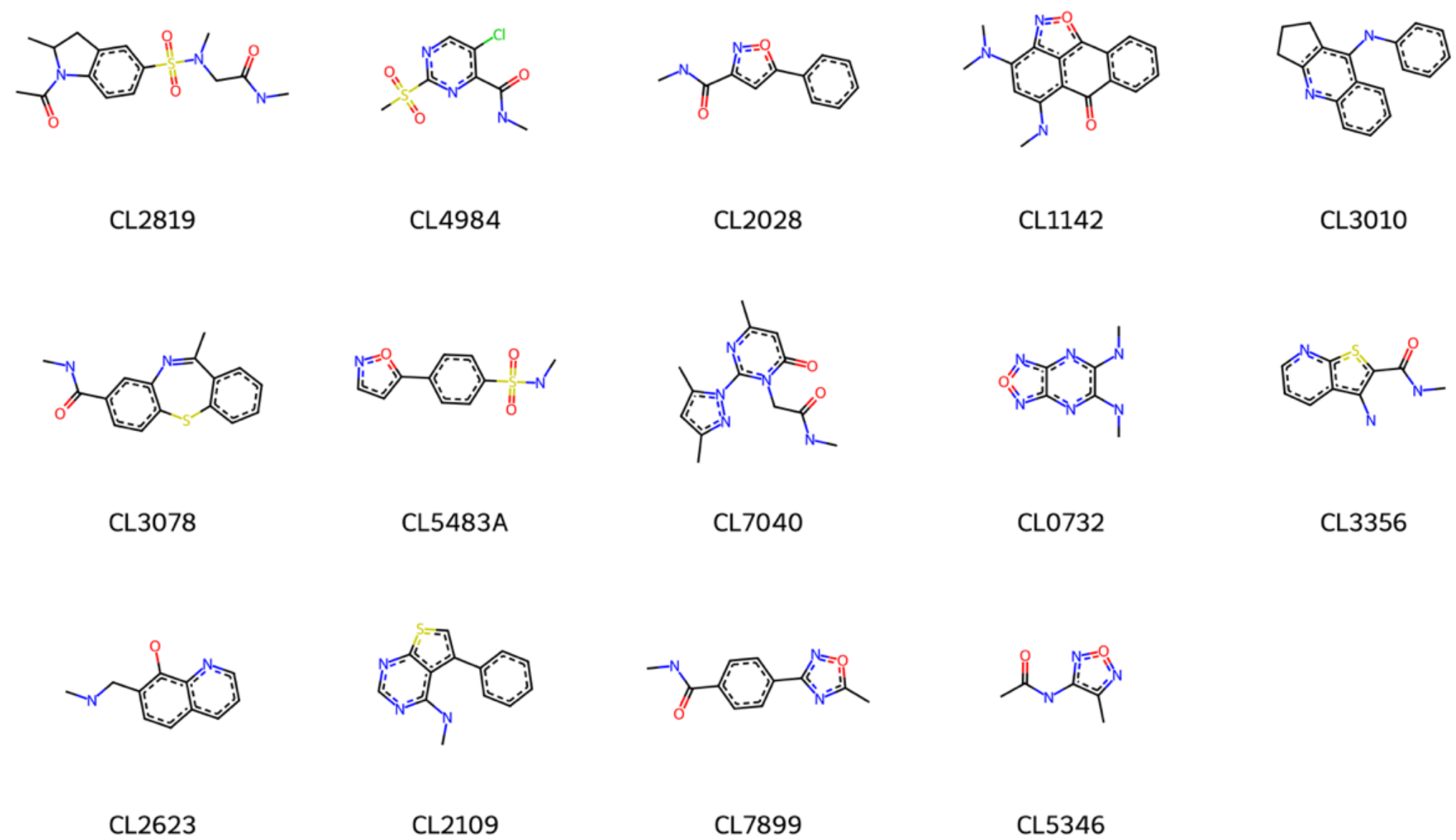

Figure R11 | Scaffold groups present in active molecules both in the HTS and the validation of prediction models.

Together, these new analyses show that we were able to identify molecules with the same growth inhibition potential than in the HTS, and also identify novel scaffolds within the predicted molecules.

We thank the Reviewer for their comment. We now include in the text explicit references to the comparable level of inhibition of the hit molecules in the validation of the predictors, and the discovery of new scaffold groups.

5. How did the authors prevent reinforcement learning moving outside the chemical domain where the scoring function is applicable?

We thank the Reviewer for this relevant observation. We did not impose explicit constraints to prevent reinforcement learning from exploring outside the chemical domain of applicability of the scoring function. Instead, the model is initialized (incepted) using active molecules from the targeted cell line (*Methods – Generative models*). This provides a chemically and biologically relevant reference space from which exploration starts, although nothing prevents exploration of other regions of the chemical universe. However, we agree that incorporating explicit domain-of-applicability modeling and out-of-distribution detection represents an important direction for future improvement, and it could help to increase the hit ratio both in the prediction-based and the generative approaches. The downside is a limited exploration of novel chemistry. We now added a specific mention to this point in the *Discussion* section.

6. When calculating IC50 values (Fig5), the concentrations used for many of the molecules do not achieve 50% inhibition making curve fitting difficult. In addition, 100% inhibition is not achieved with most of the titration series. The authors should extend the used concentrations far enough for reliable curve fitting and IC50 estimation.

We thank the Reviewer for their comment. Before addressing this point in detail, we would like to highlight that the primary objective of the experiments shown in Figure 5 is not to derive precise potency values, but rather to demonstrate a dose-response and a selective differential activity between targeted and spared cell lines. With this objective in mind, even in cases where 50% inhibition or complete cell killing is not achieved, the relative differences in response profiles between cell lines are clear, which directly addresses the biological question of selectivity that motivated these experiments.

It is also important to consider that the prediction models guiding the generation process were trained on data derived from a screening of approximately 11,000 chemically diverse compounds at a single concentration of 10uM. With this library size, the emergence of highly potent (sub-micromolar) molecules is statistically unlikely. Additionally, the predictors are not trained on quantitative potency data but binary activity labels, as the work represents a proof-of-concept for phenotype-driven generative design and stands in the hit discovery phase of the drug discovery pipeline, rather than in the lead-optimization stage. These two characteristics of the experimental design intrinsically limit the ability of the cytotoxicity classifiers or generative model to produce highly potent compounds.

Regarding concentration range, compounds were tested up to 40 uM, which is already considered a high concentration for cell-based screening assays. For reference, out of the 298 drugs in the complete GDSC2 dataset[4], only 7 of them were tested at concentrations higher than this threshold. Another example is this other high-throughput study by Lee et al[5] involving more than 10,000 drugs, in which they test concentrations ranging from 0.6nM to 46uM. Extending concentrations beyond this range is generally not informative for hit discovery, as it increasingly introduces non-specific cytotoxicity, solubility limitations, and off-target effects, rather than biologically meaningful activity. Furthermore, as mentioned previously, the objective of the study is to demonstrate the differential cytotoxicity rather than determining the exact potency of the molecules.

We have now incorporated this discussion in the *Methods* section of the revised manuscript.

7. Although testing close analogues of the predicted molecules can be OK, in many of the cases the Tanimoto-similarities are not that great and this part does not really validate the predictions in my opinion. This leaves a feeling that the project is merely a computational exercise. To get past this, could the authors test 1 or few of the predicted molecules across all PC lines to compare if they have more selectivity than the close analogues? In some of the cases it might be possible to achieve nanomolar inhibition while being specific.

We thank the Reviewer for their comment. We would like to clarify that our experimental validation does not rely solely on testing close analogues, as shown in Figure 4g., we tested both identical molecules and close analogs. Indeed, 15 of the 45 tested molecules are the same exact

generated molecules, with another 22 showing chemical Tanimoto similarities with the generated molecule > 0,95. Thus, the validation is not limited to proxy compounds, but includes direct experimental testing of generated structures. We have clarified this in the revised manuscript.

To further address the Reviewer's comment, we looked deeper into the relationship of Tanimoto similarity to generated compound and compound activity. We found 4 of the 15 identical molecules to succeed at the defined selectivity thresholds at 10uM. Figure R12 explores the relationship between generated-tested compound similarity and selective cell-line growth inhibition. While we find a weak tendency suggesting that that the tested molecules closer to the generated ones tend to more often display the intended selectivity, we also find negatives with very high chemical similarities. Although this last point is already discussed in the *Results* and *Discussion* sections of the manuscript, we now highlight the presence of generated molecules in the final validation set to avoid confusion and have included Figure R12 as Extended Data Figure 12.

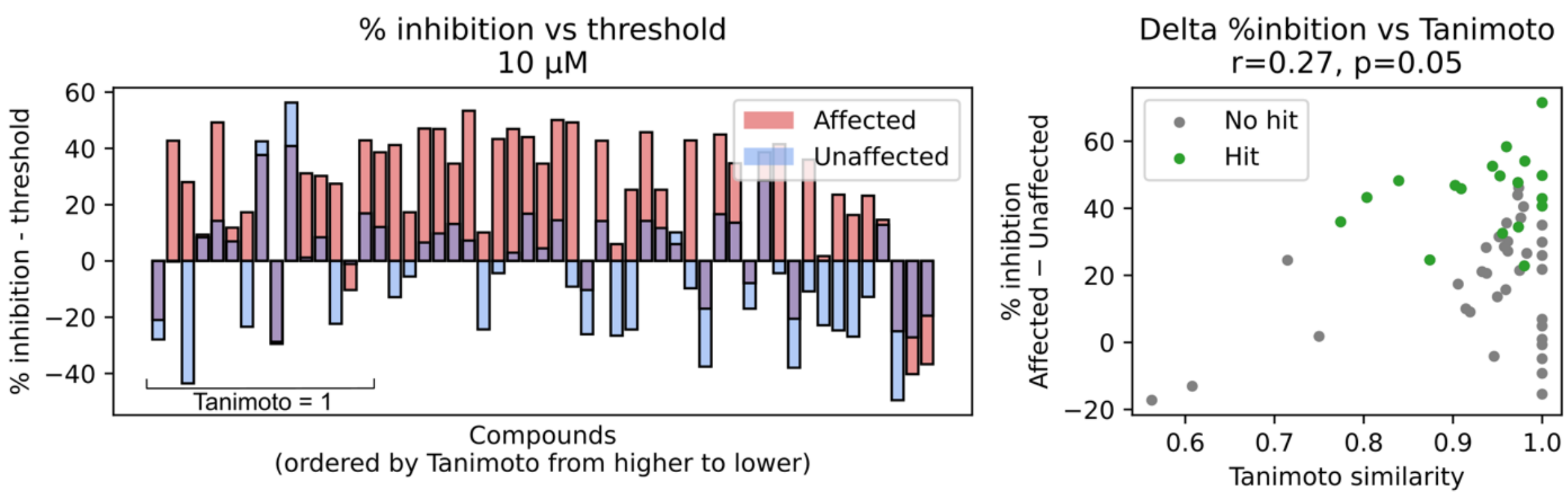

Figure R12 | Relationship between compound similarity to generated molecule and selective cell-line inhibition. Left: % inhibition vs threshold for targeted (affected) and spared (unaffected) cell lines, ordered by Tanimoto similarity to generated molecule from left to right. Right: difference in % inhibition between targeted and spared cell lines vs Tanimoto similarity to generated molecule. r = Spearman's r; p = p-value.

Minor:

8. What are "default structural alerts" and how was this implemented?

We thank the Reviewer for their question, which we failed to discuss in the initial version of the manuscript. We followed the implementation described in the REINVENT community GitHub tutorials (https://github.com/MolecularAI/ReinventCommunity) and used the default structural alert set provided therein. These alerts are defined as SMARTS patterns that encode chemically undesirable moieties or substructures, including motifs associated with toxicity, high chemical reactivity, low synthetic accessibility, or poor drug-like properties. During molecule generation, any candidate structure matching one or more of these SMARTS patterns is penalized by

reducing its reward/score, thereby discouraging the model from sampling or optimizing toward such chemistries in subsequent generations.

A breakdown of those alerts is presented in Table R2, which are often toxic, highly reactive or even mutagenic, and not desired in potential drug candidates. We now include a description of structural alerts are in the *Methods* section of the revised manuscript and have included Table R2 as Supplementary Table 12.

SMARTS pattern in structural alert	Meaning
"[*;r8]", "[*;r9]", "[*;r10]", "[*;r11]", "[*;r12]", "[*;r13]", "[*;r14]", "[*;r15]", "[*;r16]", "[*;r17]"	Ring systems with more than 8 atoms
"[#8][#8]"	Peroxide
"[#6;+]"	Positively charged carbon
"[#16][#16]"	Disulfide bond
"[#7;!n][S;!$(S(=O)=O)]"	Amine-thioether
"[#7;!n][#7;!n]"	Amine-amine
"C#C"	Alkine
"C(=[O,S])[O,S]"	Ester or thoiester
"[#7;!n][C;!$(C(=[O,N])[N,O])][#16;!s]", "[#7;!n][C;!$(C(=[O,N])[N,O])][#7;!n]", "[#7;!n][C;!$(C(=[O,N])[N,O])][#8;!o]", "[#8;!o][C;!$(C(=[O,N])[N,O])][#16;!s]", "[#8;!o][C;!$(C(=[O,N])[N,O])][#8;!o]", "[#16;!s][C;!$(C(=[O,N])[N,O])][#16;!s]"	Reactive linkers

Table R2 | Predefined structural alerts from REINVENT 3.2

9. "After the cross-validation, we used the complete set of positives as training, without a validation split, to train a final model for each cell line using CC bioactivity descriptors, as they showed superior performance." Why was this done and how was this model superiority tested?

We thank the Reviewer for their question. We apologize for the confusion, the reported superiority refers to the improved performance of models trained using CC_GLOBAL descriptors compared

to those trained with ECFP4 fingerprints, as shown in Figure 2b. This comparison was established in silico using a cross-validation framework.

Given the relatively small size of the dataset and the fact that descriptor comparison and model selection were studied during cross-validation, we combined all available data to train a final model for prospective experimental evaluation (Figure 2c). Therefore, retaining a validation subset would have unnecessarily reduced the effective training data without providing additional benefit. This strategy is a common practice in predictive modeling and machine learning workflows[6,7].

We have rewritten the sentence in the revised manuscript to clarify that the superiority refers to the in silico cross-validation results in comparison to ECFP4.

10. Odds ratio vs HTS" is not well explained. How was this metric calculated?

We thank the Reviewer for their comment, and we have now clarified the calculation of odd ratios in the *Methods* section of the revised manuscript. In brief, all odds ratios reported in this study were derived from Fisher's exact tests, comparing the fraction of active molecules in a given predicted subset against the fraction of actives in the full HTS dataset or the reference set mentioned in the corresponding section.

For instance, in Figure 2c for each prediction score range, we construct a 2×2 contingency table with the following categories:

(i) active vs inactive molecules and

(ii) HTS compounds vs model-predicted compounds within that score range.

We then compute the odds ratio using scipy.stats.fisher_exact.

Using CAPAN2 as an example, prediction scores range is 0.8–1.0, the contingency table looks like Table R3, and the odds ratio is then computed like: (3/5) / (230/10823) = 0.6 / 0.021 = 28.23

This indicates that the proportion of active compounds in the predicted set is approximately 28-fold higher than in the original HTS library, demonstrating strong enrichment of active compounds in the set predicted by the model.

	HTS	Predicted in the score range
Active	230	3
Inactive	10,823	5

Table R3 | Exemplary contingency table for odds ratio calculation (Fisher's exact test). Data corresponds to the comparison of hit ratios among the predicted molecules for CAPAN2 with a score range of 0.8-1.0 vs HTS, in the experimental validation of the cytotoxicity prediction models.

Bibliography:

1. Bertoni, M. *et al.* Bioactivity descriptors for uncharacterized chemical compounds. *Nat. Commun.* **12**, 3932 (2021).
2. Duran-Frigola, M. *et al.* Extending the small-molecule similarity principle to all levels of biology with the Chemical Checker. *Nat. Biotechnol.* **38**, 1087–1096 (2020).
3. Comajuncosa-Creus, A. *et al.* Integration of diverse bioactivity data into the Chemical Checker compound universe. Preprint at https://doi.org/10.1101/2024.12.04.626832 (2024).
4. Yang, W. *et al.* Genomics of Drug Sensitivity in Cancer (GDSC): a resource for therapeutic biomarker discovery in cancer cells. *Nucleic Acids Res.* **41**, D955-961 (2013).
5. Lee, O. W. *et al.* Cytotoxic Profiling of Annotated and Diverse Chemical Libraries Using Quantitative High-Throughput Screening. *SLAS Discov.* **25**, 9–20 (2020).
6. Liu, S. *et al.* Integrated Virtual Screening Approach Identifies New CYP19A1 Inhibitors. *J. Chem. Inf. Model.* **65**, 3529–3543 (2025).
7. Bazaga, A., Leggate, D. & Weisser, H. Genome-wide investigation of gene-cancer associations for the prediction of novel therapeutic targets in oncology. *Sci. Rep.* **10**, 10787 (2020).
