# Supplementary material for: Phenotypic AI-based design of cell-specific small molecule cytotoxics
Source: Commun Chem. 2026 May 20;9:275. doi: 10.1038/s42004-026-02071-x (PMC13457561; doi:10.1038/s42004-026-02071-x)
Supplement: Supplementary file 2 [file 42004_2026_2071_MOESM2_ESM.pdf]

Supplementary information

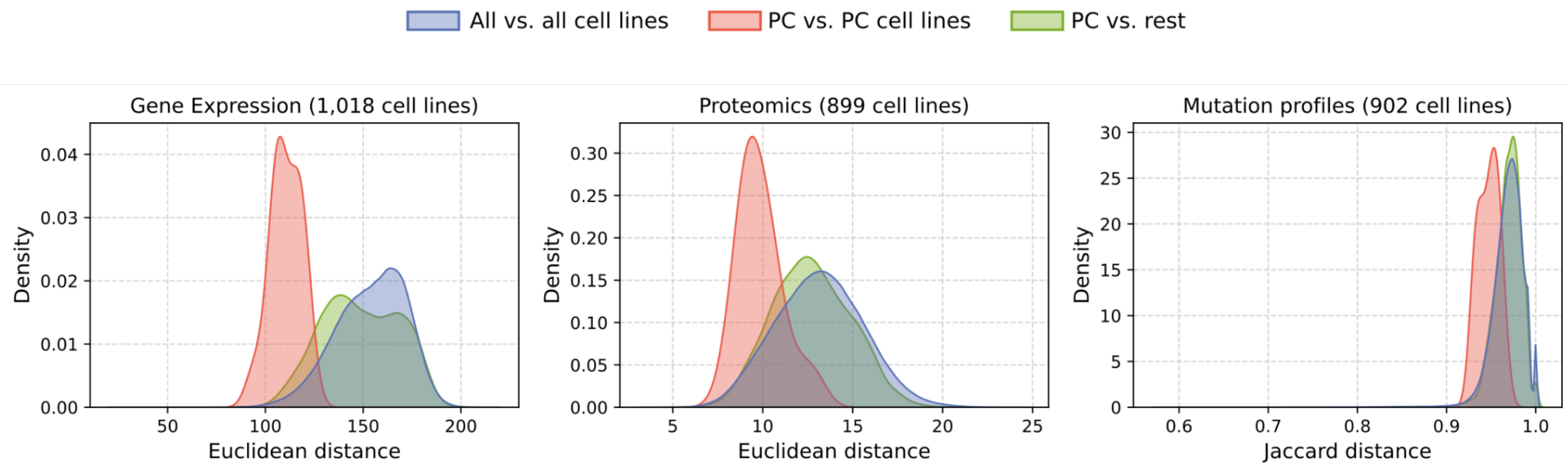

Supplementary Fig. 1 | Distance between cell lines in different biological descriptors. *PC cell lines* correspond to the 6 pancreatic cancer cell lines included in this study. *All cell lines* are the total of cell lines for which the descriptors were available. Gene expression descriptors were taken from GDSC1000[1] (RMA-processed basal expression levels for 17,737 genes measured in 1,018 cell lines). Proteomic descriptors were taken from DepMap[2] (CCLE Reverse Phase Protein Array for 144 proteins in 899 cell lines). Mutational profiles come from DrugCell[3], consisting of binary descriptors indicating mutated (1) or wild type (0) state for 3,008 genes in 902 cell lines.

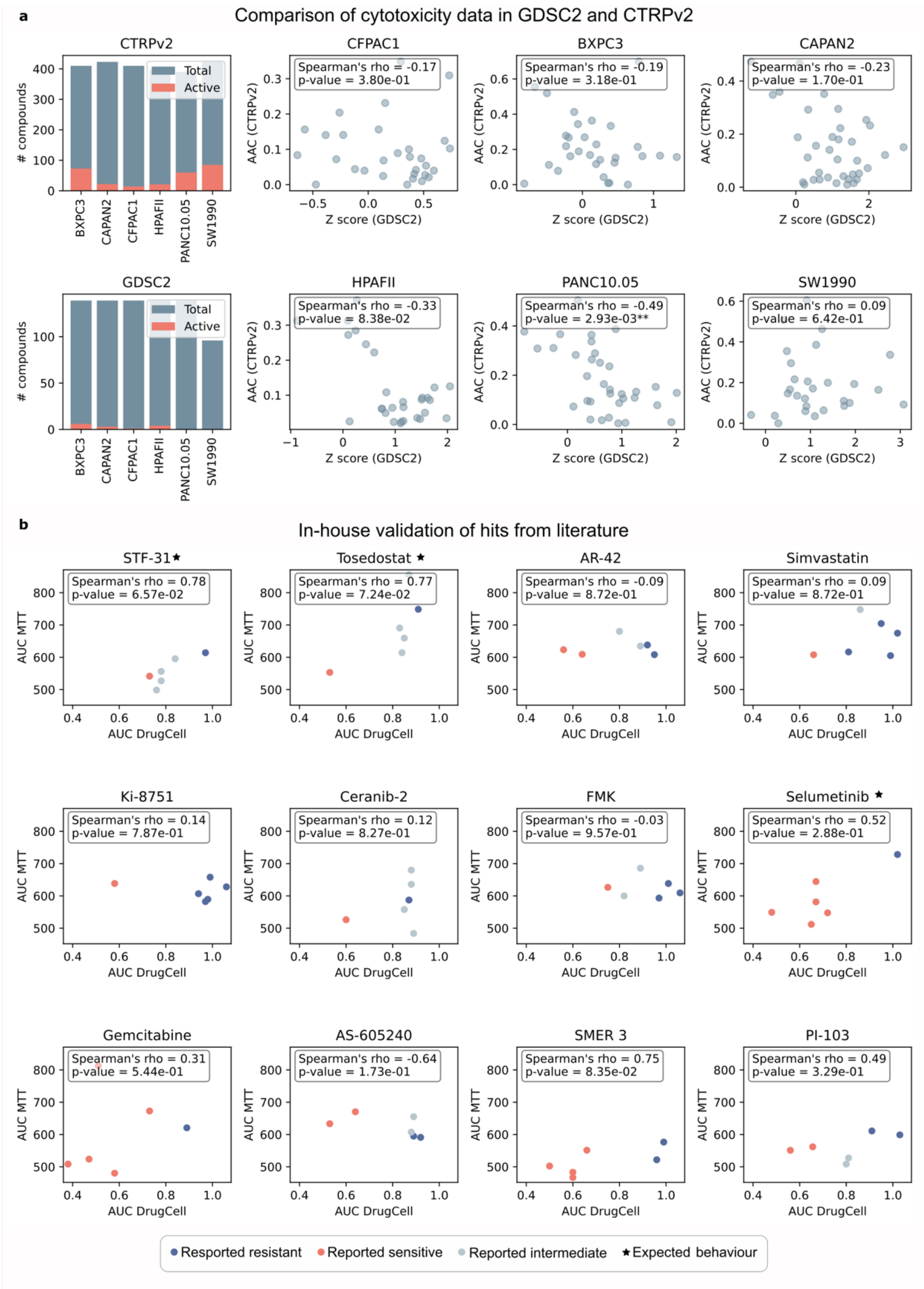

Supplementary Fig. 2 | Comparison of data in different public databases and experimental confirmation for our cell line panel. (a) On the left, the number of compounds tested and those found to be active in CTRPv2[4] (top) and GDSC2[5] (bottom) for our pancreatic cancer cell lines. The scatter plots show the activity and correlation between overlapping compounds in both databases for each cell line. AAC: area above the dose-response curve. The larger the more sensitive. Z score: sensitivity z-score (computed per compound across cell lines). The smaller the more sensitive. Stars indicate significance levels for the Spearman's correlation test, two-sided (**: p-value < 0.01). Number of molecules per cell line:

CFPAC1=30, BXPC3=30, CAPAN2=36, HPAFII=29, PANC10.05=34, and SW1990=28. **(b)** Experimental confirmation of selected molecules reported to have differential effects on our PC cell line panel. The scatter plots show the reported activity in DrugCell[3] compared to the activity we observed. AUC: area under the dose-response curve. The smaller the more sensitive. We report correlations and p-values according to Spearman's correlation test, two-sided (n=6 cell lines).

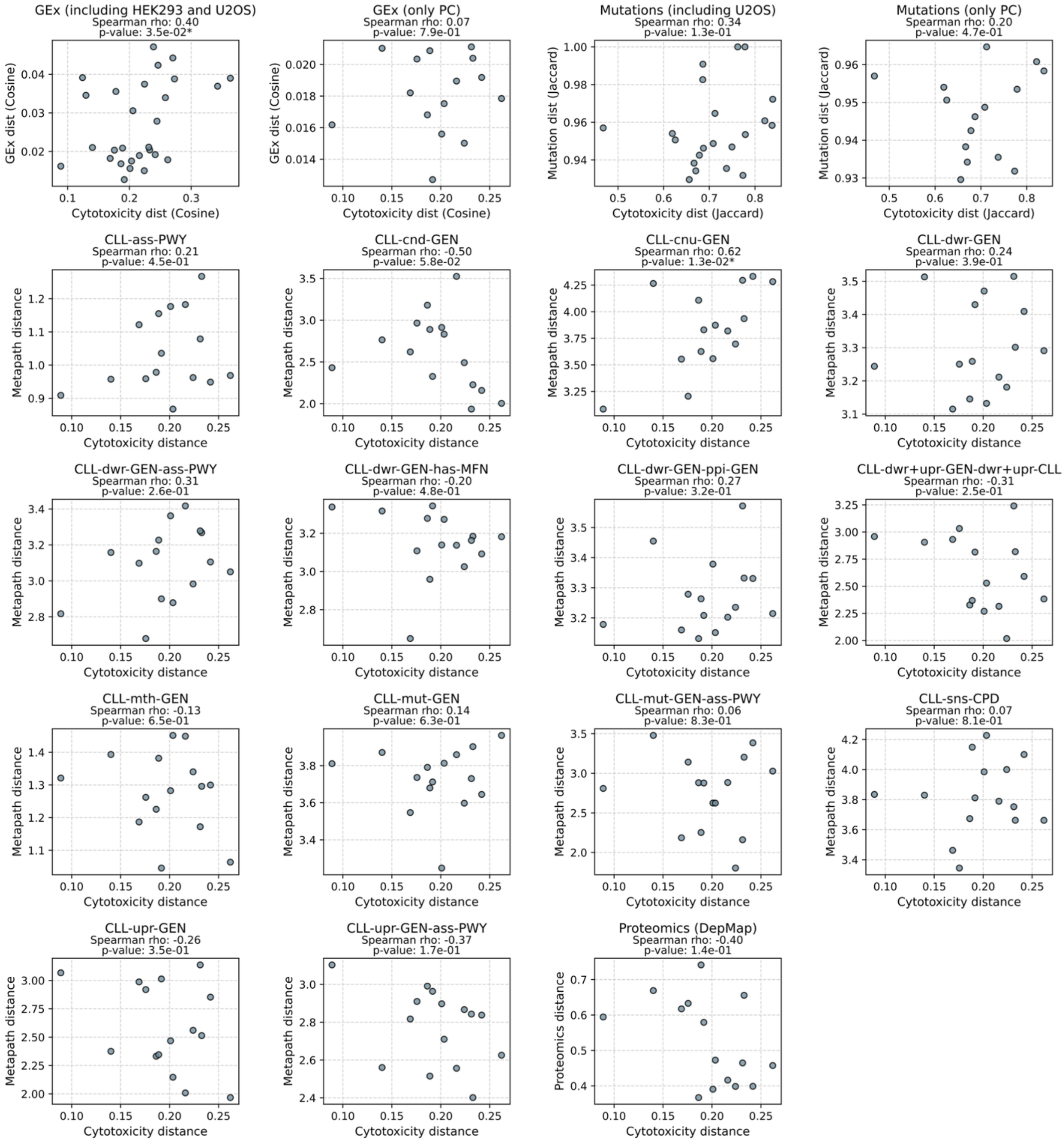

Supplementary Fig. 3 | Comparison of cell line similarities in sensitivity vs. gene expression and mutational profiles, Bioteque metapaths and proteomics. We examined the Spearman's correlation between cell line distances in cytotoxicity and distances in gene expression (GEx) profiles (in-house), mutational profiles from DrugCell[39], Bioteque metapaths[6] and proteomics data from DepMap[2], focusing on the six pancreatic cancer (PC) cell lines of interest (see *Methods*). In the case of GEx and mutational profiles, we show separate plots including the U2OS and HEK293 cell lines for comparison. Stars indicate the level of significance (*: p-value < 0.05). Sample size = 15 for all comparisons except for Mutations (including U2OS) with sample size = 21 and GEx (including HEK293 and U2OS), with sample size = 28. Sample size corresponds to pairwise comparisons of 6, 7 or 8 cell lines respectively. See Supplementary Table 11 for Bioteque metapath description.

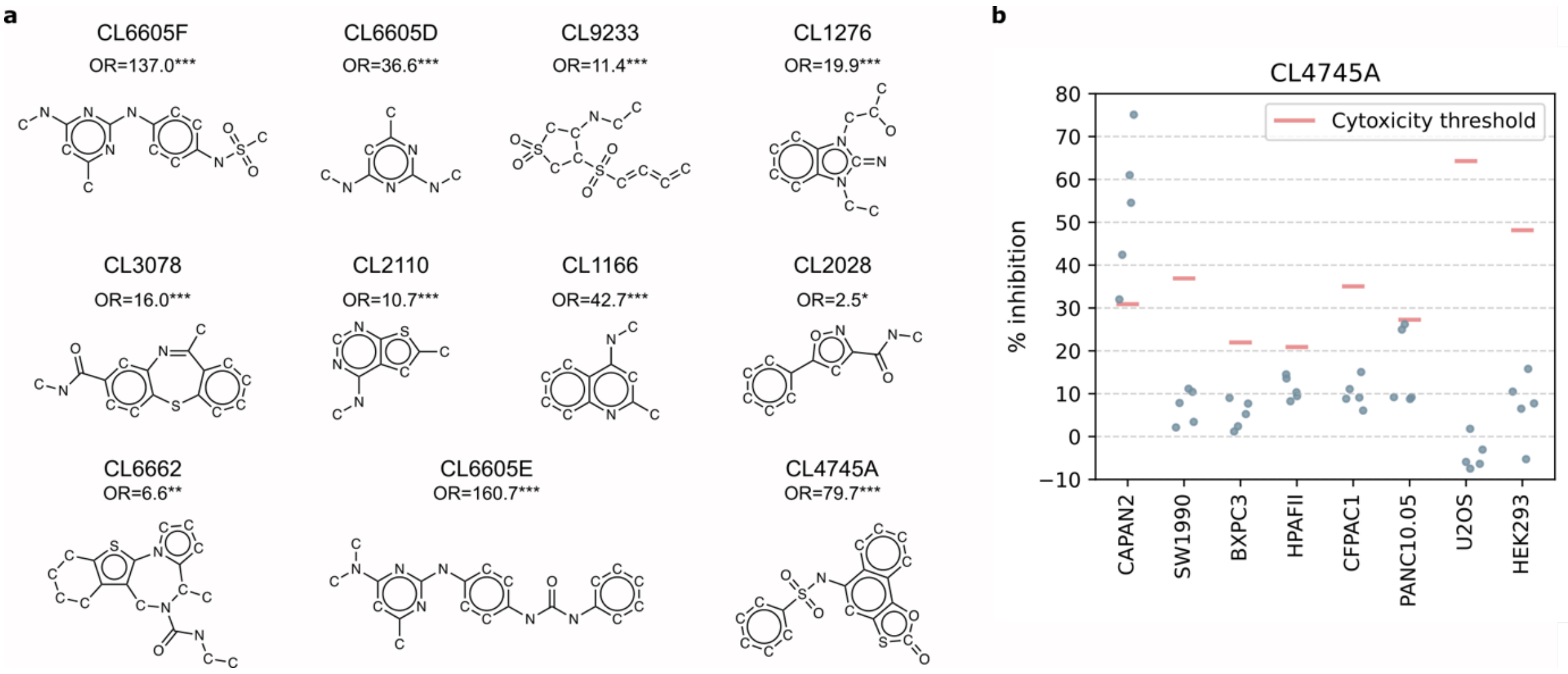

Supplementary Fig. 4 | Scaffolds with >5 active molecules confirmed after HTS. (a) Molecular structures of the scaffolds with more than five active molecules confirmed in the high-throughput screening (HTS). OR: odds ratio from a Fisher's exact test (two-sided) comparing ratio of active molecules containing those scaffolds compared to the whole HTS (n=11,053). Group sizes (n) for molecules having the given scaffold are: CL6605F=28, CL6605D=26, CL9233=29, CL1276=9, CL3078=18, CL2110=20, CL1166=11, CL2028=52, CL6662=17, CL6605E=11, CL4745A=6. *, **, ***: p-value<0.05, 0.01, 0.001. **(b)** % inhibition per cell line for the five molecules containing the scaffold CL4745A, which showed specificity to CAPAN2.

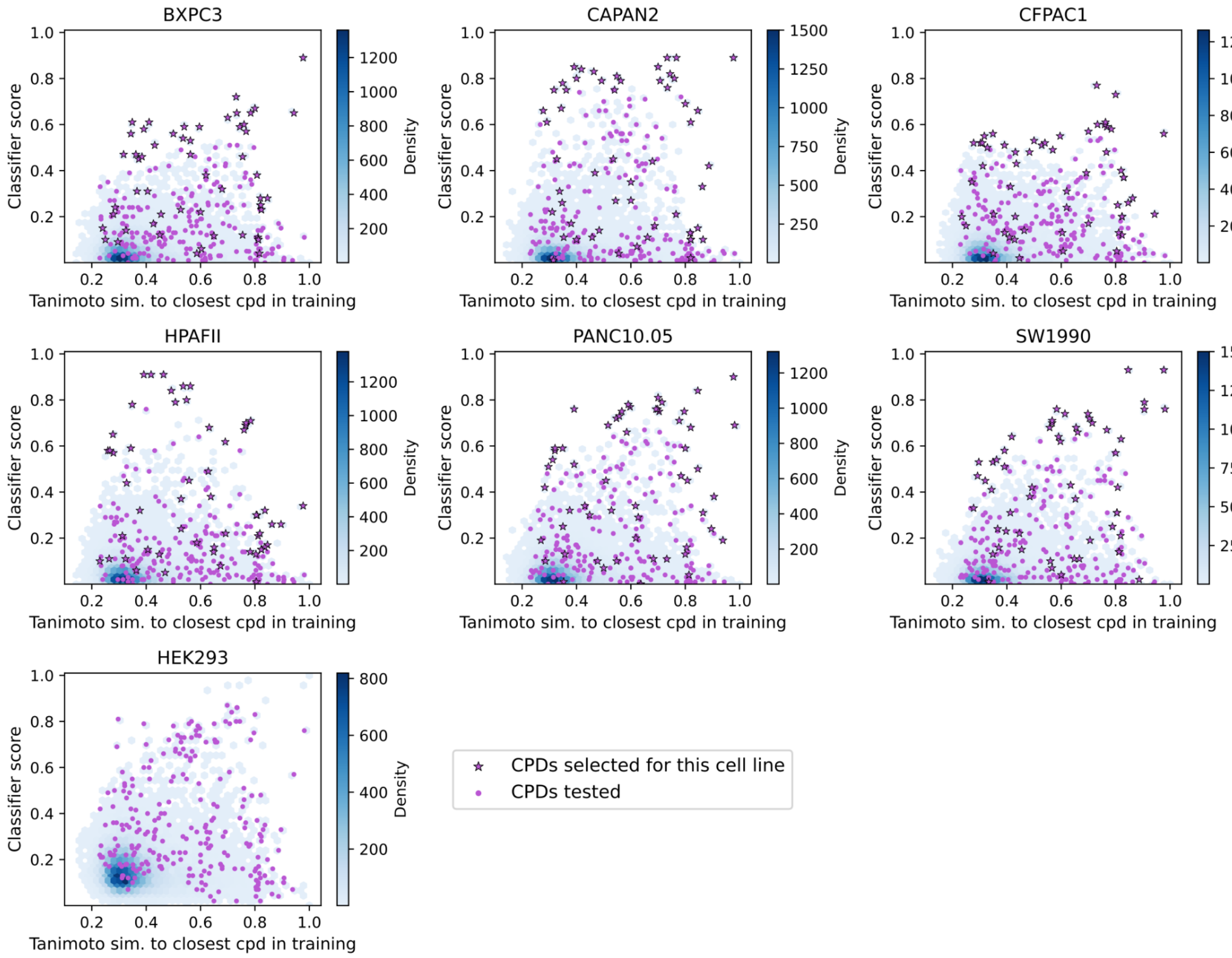

Supplementary Fig. 5 | Tanimoto similarity to training compounds vs predicted score for compounds selected for validation of cytotoxicity predictors. To experimentally validate the cytotoxicity predictors, predictions were made for the entire in-house library, and a subset of compounds was selected based on varying Tanimoto similarity scores to the closest molecules in the training dataset and different classifier-predicted scores (see *Methods*). The plot shows, for each cell line, the Tanimoto similarities and predicted scores for the entire in-house library (blue) and highlights the selected compounds (pink). Star markers denote compounds specifically selected for the given cell line.

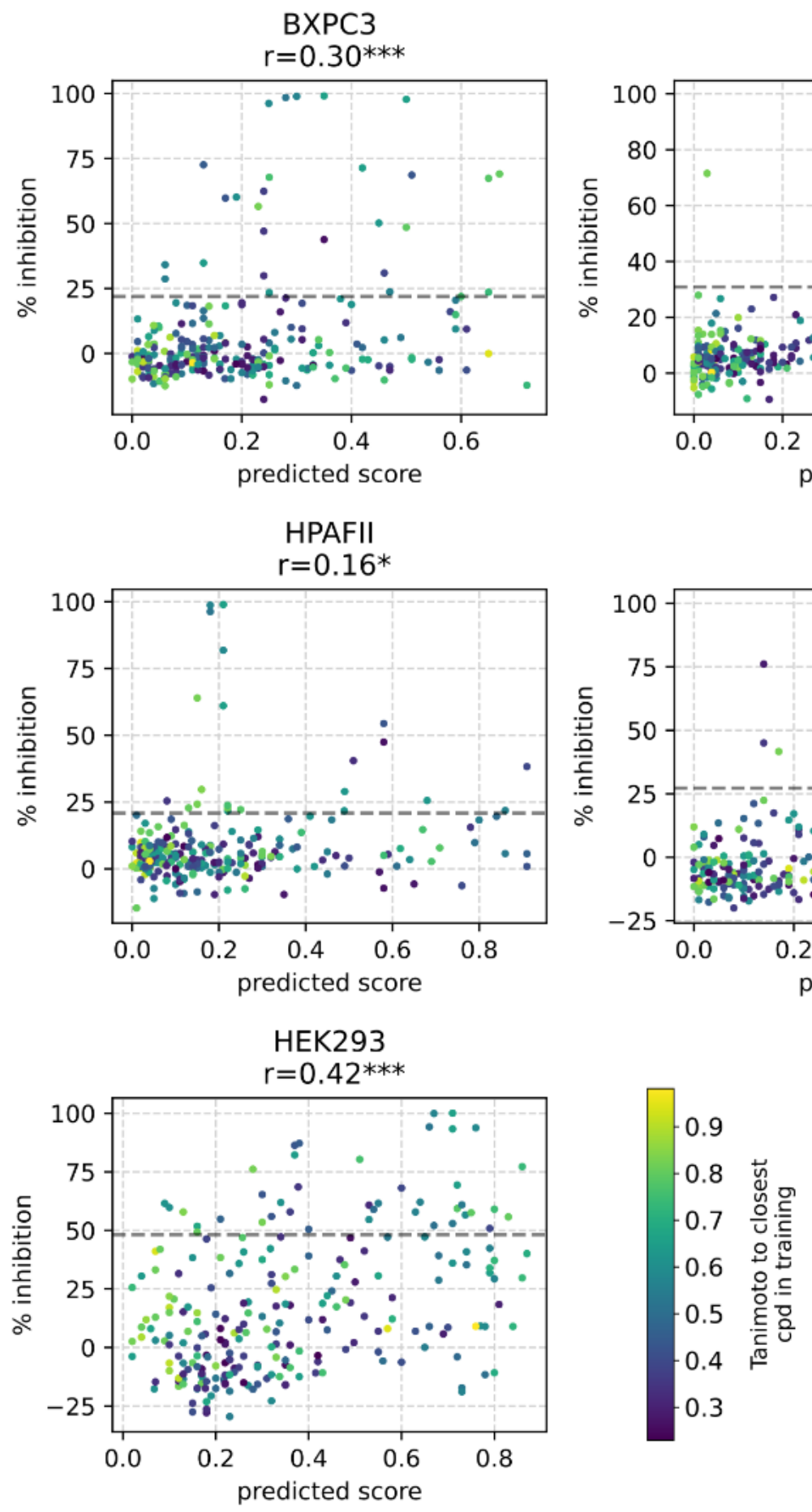

Supplementary Fig. 6 | Experimental validation of the cytotoxicity predictors. Predicted scores versus % inhibition observed in the experimental validation of the cytotoxicity predictors for each cell line. Data points are colored based on Tanimoto similarity to the closest compound in the training dataset. Dashed lines represent the cytotoxicity thresholds. r shows Pearson correlation (two-sided) between axes. *, **, ***: p-value < 0.05, 0.01, 0.001. n=244 molecules.

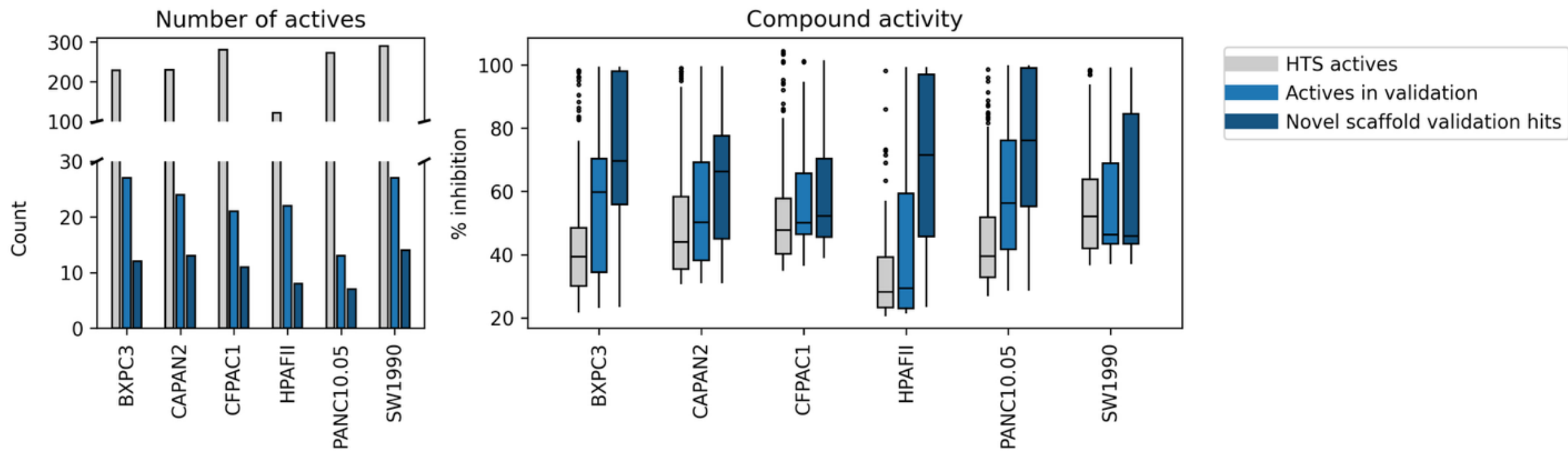

Supplementary Fig. 7 | Comparison of the inhibitory potential of HTS and validation hit compounds. The left panel shows the number of active molecules identified in both screenings, alongside the number of validated hits possessing novel scaffolds in comparison to the original HTS actives. The right panel shows the corresponding cytotoxic activity (%inhibition) for these molecules. Boxplot elements: center line, median; box limits, upper and lower quartiles; whiskers, 1.5x interquartile range; points, outliers.

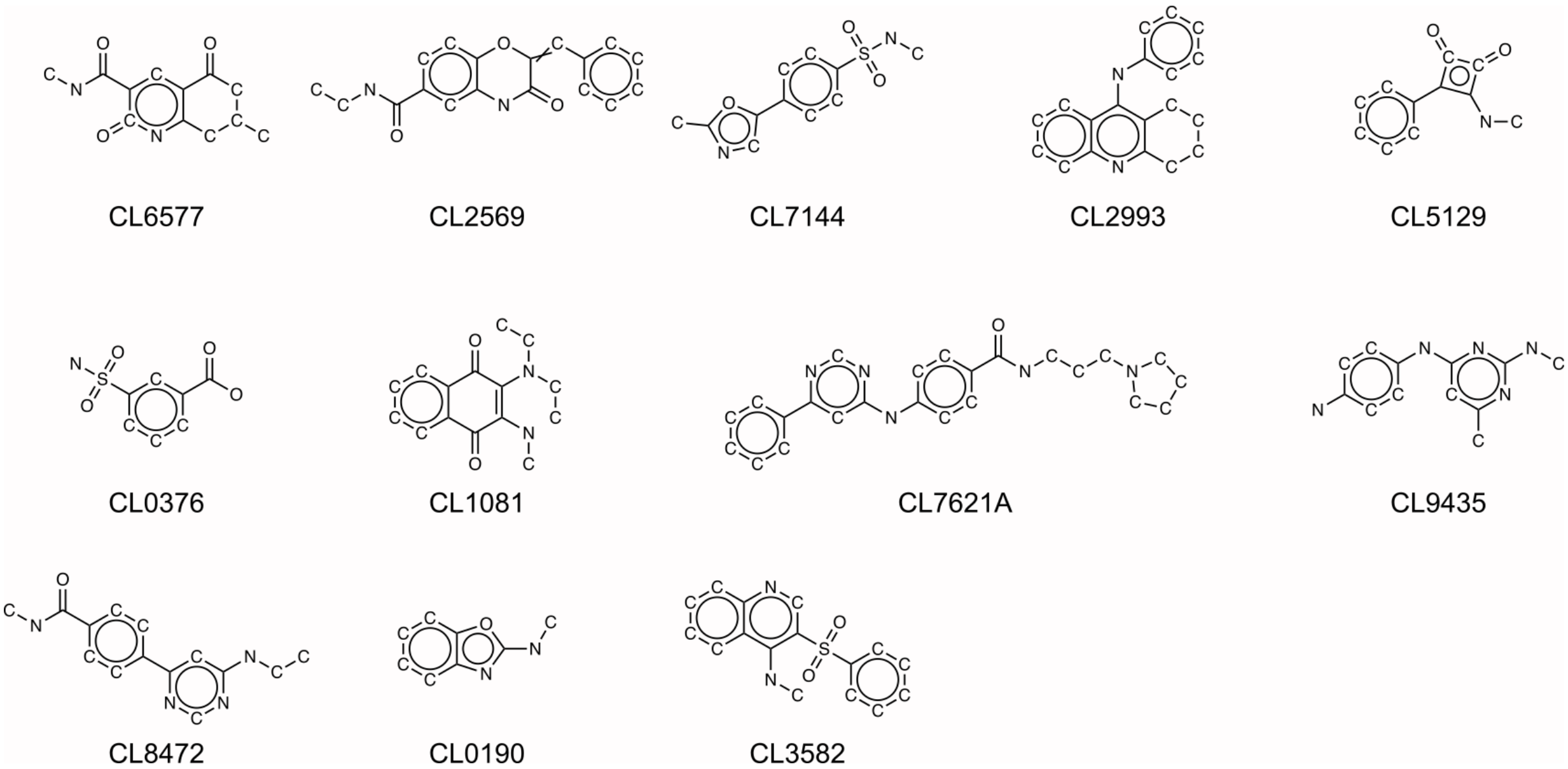

Supplementary Fig. 8 | New scaffold groups in active molecules identified during validation of prediction models.

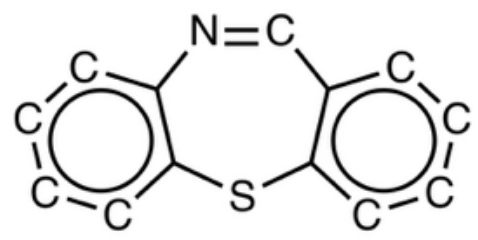

Supplementary Fig. 9 | Repetitive substructure in the molecular generation process. Both for the exercises BXPC3 vs CAPAN2 and BXPC3 vs HEK293 we found this substructure to be extremely prevalent in the proposed designs, thus the generative cycle was run again penalising the molecules containing this motif.

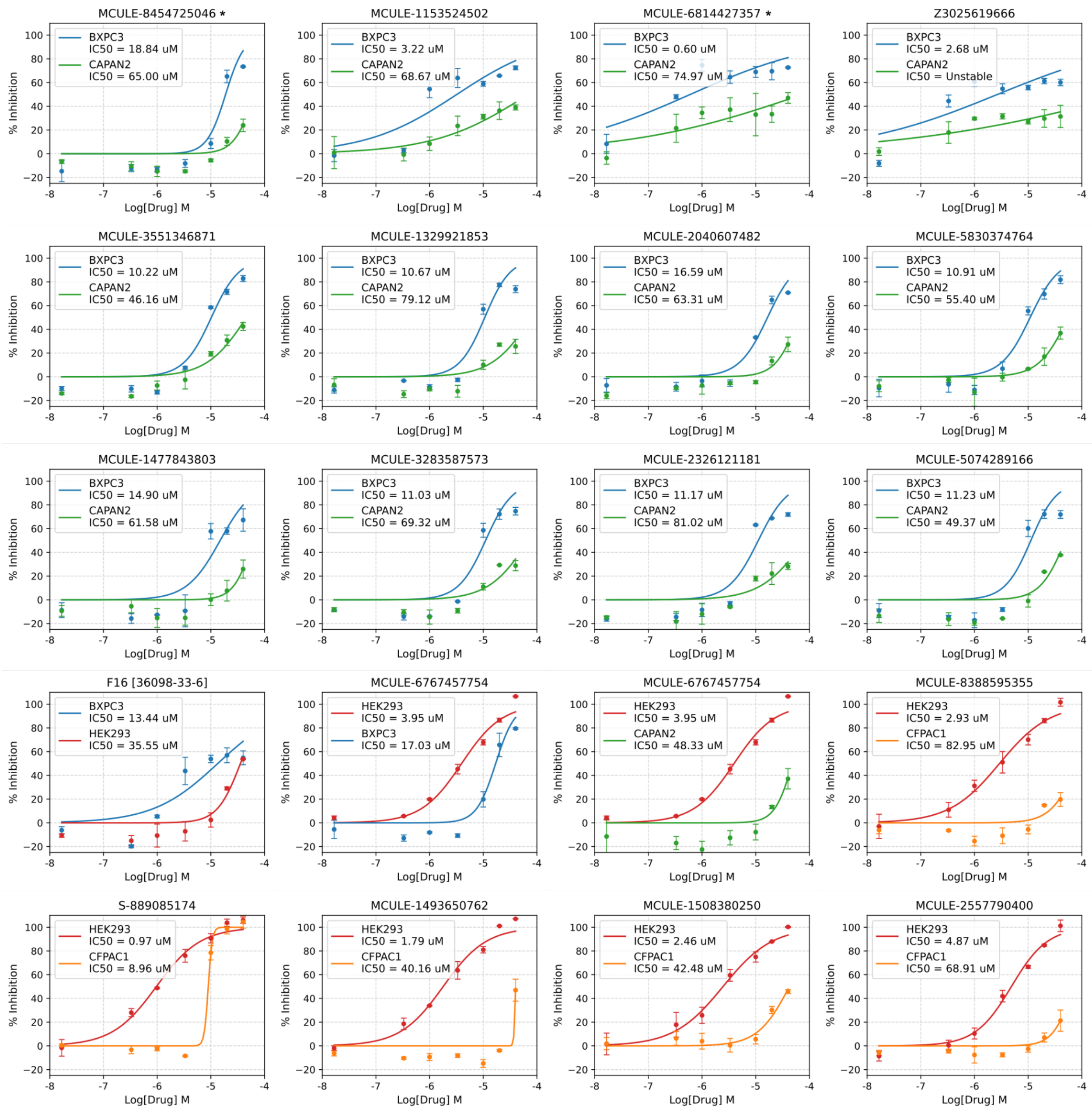

Supplementary Fig. 10 | Dose-response curves for the hits found through the generative approach. Stars indicate compounds that did not pass the cytotoxicity thresholds at 10 μM in the dose-response assay. Error bars show standard deviation between replicates.

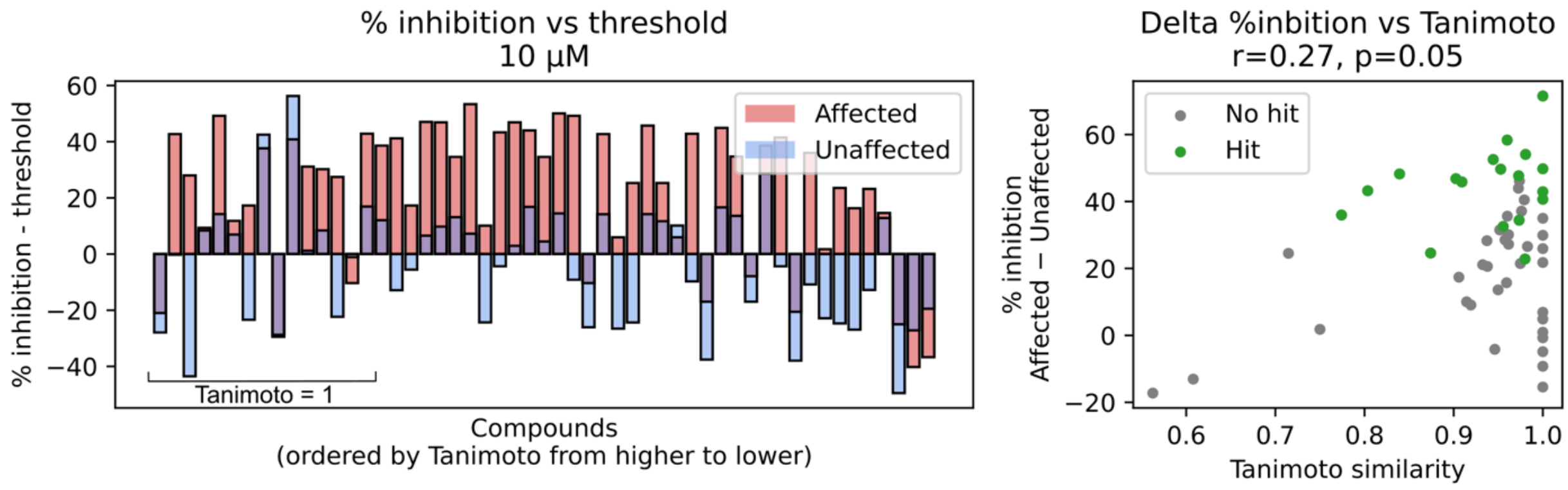

Supplementary Fig. 11 | Relationship between compound similarity to generated molecule and selective cell-line inhibition. Left: % inhibition vs threshold for targeted (affected) and spared (unaffected) cell lines, ordered by Tanimoto similarity to generated molecule from left to right. Right: difference in % inhibition between targeted and spared cell lines vs Tanimoto similarity to generated molecule. r = Spearman's r, two sided; p = p-value, n = 53.

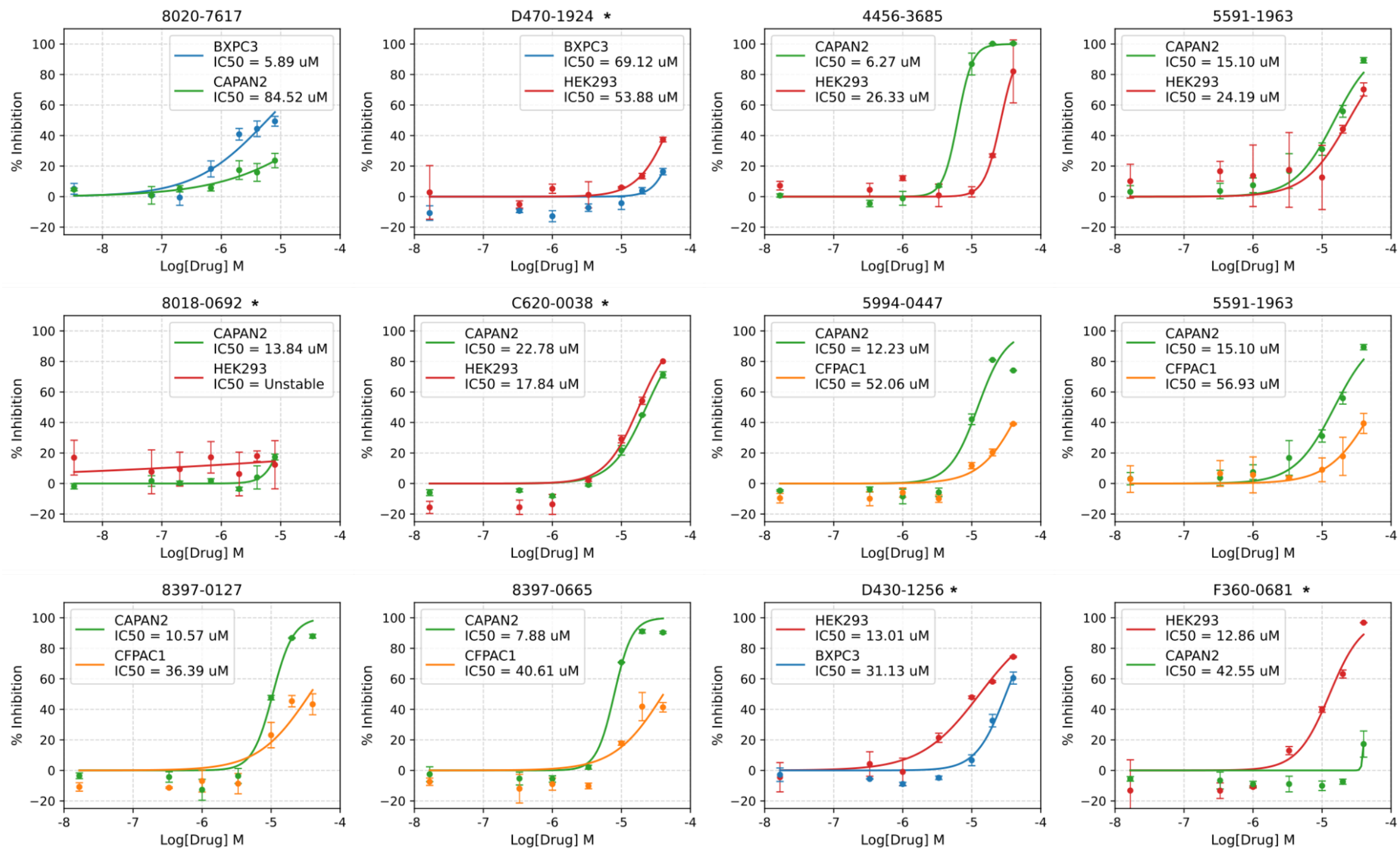

Supplementary Fig. 12 | Dose-response curves for the hits found through the prediction-only approach. Stars indicate compounds that did not pass the cytotoxicity thresholds at 10 µM in the dose-response assay. Error bars show standard deviation between replicates.

Name	Histology	Tumor Source	Mutant Gene	Zygosity	Gene Sequence	Protein Sequence
CAPAN2	Adenocarcinoma	primary	KRAS	heterozygous	c.35G>T	p.G12V
PANC10.05	Adenocarcinoma	primary	KRAS<br>TP53	heterozygous<br>heterozygous	c.35G>A<br>c.764T>A	p.G12D<br>p.I255N
CFPAC1	Ductal adenocarcinoma	metastasis, liver	KRAS<br>SMAD4<br>TP53	heterozygous<br>homozygous<br>homozygous	c.35G>T<br>c.1_1659del1659<br>c.724T>C	p.G12V<br>p.0?<br>p.C242R
HPAFII	Adenocarcinoma	metastasis, ascites	CDKN2A<br>KRAS<br>TP53	homozygous<br>heterozygous<br>homozygous	c.85_101del17<br>c.35G>A<br>c.451C>T	p.R29fs*9<br>p.G12D<br>p.P151S
SW1990	Adenocarcinoma	metastasis, spleen	CDKN2A<br>KRAS	homozygous<br>homozygous	c.1_471del471<br>c.35G>A	p.0?<br>p.G12D
BXPC3	Adenocarcinoma	primary	CDKN2A<br>MAP2K4<br>SMAD4<br>TP53	homozygous<br>homozygous<br>homozygous<br>homozygous	c.1_471del471<br>c.1041_1200del160<br>c.1_1659del1659<br>c.659A>G	p.0?<br>p.?<br>p.0?<br>p.Y220C
AsPC-1	Adenocarcinoma	metastasis, ascites	CDKN2A<br>FBXW7<br>KRAS<br>MAP2K4<br>TP53	homozygous<br>heterozygous<br>homozygous<br>homozygous<br>homozygous	c.233_234delTC<br>c.1393C>T<br>c.35G>A<br>c.1-?_393+?del<br>c.403delT	p.L78fs*41<br>p.R465C<br>p.G12D<br>p.?<br>p.C135fs*35

Supplementary Table 1 | Description of pancreatic cancer cell lines (from ATCC® TCP-1026 Pancreatic Cancer Panel).

Database	# Compounds (CPD)	# Cell lines (CLL)	CPD-CLL pairs	# Pancreatic cell lines	# Cell lines of interest (9)	Info
GDSC2	198	809	135,242	31	8	IC50<br>IC50 Z-score<br>AUC<br>Sensitive/resistant classification
NCI-60	23,441	60	1,442,820	0	0	Z-scored IC50
CTRPv2	484	887	343,606	29	8	AAC
DrugCell	684	1,224	509,294	42	8	AUC

Supplementary Table 2 | Cytotoxicity data in public databases.

Cell line	Odds ratio	p-value
CAPAN2	2.61	6e-7***
SW1990	2.85	6e-10***
BXPC3	3.31	7e-11***
HPAFII	2.64	2e-4***
CFPAC1	3.20	5e-12***
PANC10.05	3.17	2e-11***

Supplementary Table 3 | Enrichment of hits within the nearest neighbors from reported actives in literature vs rest of molecules tested (HTS). ***: p-value < 0.001. Fisher's exact test, two-sided. Non-neighbors n=10,182; neighbors n=871 neighbors.

<table>
<tr><th>Exercises</th><th>Run type</th><th>Phase</th><th># Epochs</th><th>Diversity filter</th><th>Scoring function* components (weight)</th></tr>
<tr><td>BXPC3 vs CAPAN2
CAPAN2 vs HEK293</td><td>Reinforcement learning</td><td>-</td><td>2,000</td><td>Scaffold Similarity</td><td>- CLL1** classifier (5)
- CLL2*** classifier (2)
- Molecular weight (2)
- Default structural alerts (1)</td></tr>
<tr><td rowspan="2">BXPC3 vs CAPAN2
BXPC3 vs HEK293
CAPAN2 vs HEK293
CAPAN2 vs CFPAC1
HEK293 vs BXPC3
HEK293 vs CAPAN2
HEK293 vs CFPAC1</td><td rowspan="2">Curriculum learning</td><td>Curriculum</td><td>Up to 3,000</td><td>Scaffold Similarity</td><td>- CLL1 classifier (5)
- Molecular weight (2)
- Default structural alerts (1)</td></tr>
<tr><td>Production</td><td>2,000</td><td>Scaffold Similarity</td><td>- CLL1 classifier (5)
- CLL2 classifier (2)
- Molecular weight (2)
- Default structural alerts (1)</td></tr>
<tr><td rowspan="2">BXPC3 vs CAPAN2
BXPC3 vs HEK293</td><td rowspan="2">Curriculum learning (unwanted moiety)</td><td>Curriculum</td><td>Up to 3,000</td><td>Scaffold Similarity</td><td>- CLL1 classifier (5)
- Molecular weight (2)
- Default structural alerts (1)</td></tr>
<tr><td>Production</td><td>2,000</td><td>Scaffold Similarity</td><td>- CLL1 classifier (5)
- CLL2 classifier (2)
- Molecular weight (2)
- Default structural alerts + unwanted moiety (1)</td></tr>
</table>

Supplementary Table 4 | Parameters overview of REINVENT runs. *The aggregation method for the scoring function was ‘custom product’ for all the exercises. **CLL1: targeted cell line. ***CLL2: spared cell line. The rest of the tunable parameters were kept as default.

Exercise	HTS confirmation		Generative models				IRB library predictions		
	Tested	Active	Unique	High scoring (% over unique)	Tested	Active	Total	High scoring – Tested	Active
BXPC3 vs CAPAN2	751	84	5,111	137 (2.68%)	16	12	133,672	7	1
BXPC3 vs HEK293	751	54	5,067	47 (0.93%)	22	1	133,672	5	1
CAPAN2 vs HEK293	751	82	18,707	100 (0.53%)	1	0	133,672	27	4
CAPAN2 vs CFPAC1	751	84	9,235	121 (1.31%)	1	0	133,672	17	4
HEK293 vs BXPC3	751	78	8,943	38 (0.42%)	1	1	133,672	3	1
HEK293 vs CAPAN2	751	100	4,603	40 (0.87%)	1	1	133,672	2	1
HEK293 vs CFPAC1	751	79	8,692	60 (0.69%)	11	5	133,672	4	0

Supplementary Table 5 | Number of molecules generated, high-scoring, tested and active throughout the project. The table reports the number of molecules within each category in the HTS, generative and prediction pipelines.

Metapath abbreviation	Metapath full name	Dataset*
CLL-ass-PWY	Cell associates Pathway	Cellosaurus-Cosmic
CLL-cnd-GEN	Cell copy number down Gene	GDSC
CLL-cnu-GEN	Cell copy number up Gene	CCLE_hmz
CLL-dwr-GEN	Cell downregulates Gene	GDSC
CLL-dwr-GEN-ass-PWY	Cell downregulates Gene (that) associates Pathway	GDSC - Reactome
CLL-dwr-GEN-has-MFN	Cell downregulates Gene (that) has Molecular Function	GDSC - GO
CLL-dwr-GEN-ppi-GEN	Cell downregulates Gene (that) protein-protein interacts Gene	GDSC - STRING
CLL-dwr+upr-GEN-dwr+upr-CLL	Cell downregulates or upregulates Gene	GDSC - GDSC
CLL-mth-GEN	Cell methylates Gene	GDSC
CLL-mut-GEN	Cell mutation Gene	CCLE
CLL-mut-GEN-ass-PWY	Cell mutation Gene (that) associates Pathway	GDSC - Reactome
CLL-sns-CPD	Cell sensitive Compound	Drugcell
CLL-upr-GEN	Cell upregulates Gene	GDSC
CLL-upr-GEN-ass-PWY	Cell upregulates Gene (that) associates Pathway	GDSC - Reactome

Supplementary Table 6 | Table with abbreviation and meaning of the 11 Bioteque metapaths used as cell line descriptors to compare to cell line sensitivity (Supplementary Fig. 3). *Dataset names as they appear in the Bioteque[6] web resource, where we downloaded the descriptors from.

SMARTS pattern in structural alert	Meaning
"[*;r8]", "[*;r9]", "[*;r10]", "[*;r11]", "[*;r12]", "[*;r13]", "[*;r14]", "[*;r15]", "[*;r16]", "[*;r17]"	Ring systems with more than 8 atoms
"[#8][#8]"	Peroxide
"[#6;+]"	Positively charged carbon
"[#16][#16]"	Disulfide bond
"[#7;!n][S;!$(S(=O)=O)]"	Amine-thioether
"[#7;!n][#7;!n]"	Amine-amine
"C#C"	Alkine
"C(=[O,S])[O,S]"	Ester or thoiester
"[#7;!n][C;!$(C(=[O,N])[N,O])][#16;!s]", "[#7;!n][C;!$(C(=[O,N])[N,O])][#7;!n]", "[#7;!n][C;!$(C(=[O,N])[N,O])][#8;!o]", "[#8;!o][C;!$(C(=[O,N])[N,O])][#16;!s]", "[#8;!o][C;!$(C(=[O,N])[N,O])][#8;!o]", "[#16;!s][C;!$(C(=[O,N])[N,O])][#16;!s]"	Reactive linkers

Supplementary Table 7 | Predefined structural alerts from REINVENT 3.2

Supplementary references

1. Iorio, F. *et al.* A Landscape of Pharmacogenomic Interactions in Cancer. *Cell* **166**, 740–754 (2016).
2. DepMap, B. DepMap 24Q4 Public. 30825074613 Bytes Figshare+ https://doi.org/10.25452/FIGSHARE.PLUS.27993248.V1 (2024).
3. Kuenzi, B. M. *et al.* Predicting Drug Response and Synergy Using a Deep Learning Model of Human Cancer Cells. *Cancer Cell* **38**, 672-684.e6 (2020).
4. Rees, M. G. *et al.* Correlating chemical sensitivity and basal gene expression reveals mechanism of action. *Nat. Chem. Biol.* **12**, 109–116 (2016).

5. Yang, W. *et al.* Genomics of Drug Sensitivity in Cancer (GDSC): a resource for therapeutic biomarker discovery in cancer cells. *Nucleic Acids Res.* **41**, D955-961 (2013).
6. Fernández-Torras, A., Duran-Frigola, M., Bertoni, M., Locatelli, M. & Aloy, P. Integrating and formatting biomedical data as pre-calculated knowledge graph embeddings in the Bioteque. *Nat. Commun.* **13**, 5304 (2022).
