# Supplementary material for: Phenotypic AI-based design of cell-specific small molecule cytotoxics
Source: Commun Chem. 2026 May 20;9:275. doi: 10.1038/s42004-026-02071-x (PMC13457561; doi:10.1038/s42004-026-02071-x)
Supplement: Supplementary file 3 [file 42004_2026_2071_MOESM3_ESM.pdf]

Description of Additional Supplementary Files:

File name: Supplementary Data 1

Description: Evaluation of selective cytotoxicity in previously reported drugs.

The table shows dose–response curves for 12 drugs with selective activity toward one or more cell lines in our panel, as reported in DrugCell.

File name: Supplementary Data 2

Description: High-throughput cytotoxicity screening and hit confirmation.

The table includes the final training set derived from this data, which we used to model cytotoxicity prediction, and the posterior screening of one of the plates from the HTS on HEK293.

File name: Supplementary Data 3

Description: Gene expression data.

The table reports RNA-seq raw counts per cell line.

File name: Supplementary Data 4

Description: Experimental validation of cytotoxicity prediction models.

File name: Supplementary Data 5

Description: Experimental validation of generated molecules vs IRB library predictions.

The table reports initial validations at 1 and 10 μM and subsequent dose–response curves for the identified hits.
