# Supplementary material for: Phenotypic AI-based design of cell-specific small molecule cytotoxics
Source: Commun Chem. 2026 May 20;9:275. doi: 10.1038/s42004-026-02071-x (PMC13457561; doi:10.1038/s42004-026-02071-x)
Supplement: Supplementary file 4 [file 42004_2026_2071_MOESM9_ESM.pdf]

nature portfolio

Corresponding author(s): Patrick Aloy

Last updated by author(s): Apr 20, 2026

Reporting Summary

Nature Portfolio wishes to improve the reproducibility of the work that we publish. This form provides structure for consistency and transparency in reporting. For further information on Nature Portfolio policies, see our Editorial Policies and the Editorial Policy Checklist.

Statistics

For all statistical analyses, confirm that the following items are present in the figure legend, table legend, main text, or Methods section.

n/a	Confirmed	
☐	☒	The exact sample size (*n*) for each experimental group/condition, given as a discrete number and unit of measurement
☐	☒	A statement on whether measurements were taken from distinct samples or whether the same sample was measured repeatedly
☐	☒	The statistical test(s) used AND whether they are one- or two-sided<br>*Only common tests should be described solely by name; describe more complex techniques in the Methods section.*
☒	☐	A description of all covariates tested
☐	☒	A description of any assumptions or corrections, such as tests of normality and adjustment for multiple comparisons
☐	☒	A full description of the statistical parameters including central tendency (e.g. means) or other basic estimates (e.g. regression coefficient) AND variation (e.g. standard deviation) or associated estimates of uncertainty (e.g. confidence intervals)
☐	☒	For null hypothesis testing, the test statistic (e.g. *F*, *t*, *r*) with confidence intervals, effect sizes, degrees of freedom and *P* value noted<br>*Give P values as exact values whenever suitable.*
☒	☐	For Bayesian analysis, information on the choice of priors and Markov chain Monte Carlo settings
☒	☐	For hierarchical and complex designs, identification of the appropriate level for tests and full reporting of outcomes
☐	☒	Estimates of effect sizes (e.g. Cohen's *d*, Pearson's *r*), indicating how they were calculated

Our web collection on statistics for biologists contains articles on many of the points above.

Software and code

Policy information about availability of computer code

Data collection	Experimental data were collected using the EnVision system (Revvity), recording the signal from each well across all assay plates. Positional matching was subsequently performed between the tested compounds and the library registered in Dotmatics to ensure correct compound identification and accurate data annotation.
Data analysis	Data analysis was carried out using Studies, and Vortex (Dotmatics) for processing and visualization. The percentage of inhibition (% inhibition) was calculated for each compound, along with the corresponding quality control metrics. All plates yielded Z′ factor values above 0.5, indicating a robust assay with adequate signal separation between positive and negative controls.<br>The rest of the analysis in the paper were done in Python3, and the code is available on GitHub.

For manuscripts utilizing custom algorithms or software that are central to the research but not yet described in published literature, software must be made available to editors and reviewers. We strongly encourage code deposition in a community repository (e.g. GitHub). See the Nature Portfolio guidelines for submitting code & software for further information.

Data

Policy information about availability of data

All manuscripts must include a data availability statement. This statement should provide the following information, where applicable:

- Accession codes, unique identifiers, or web links for publicly available datasets
- A description of any restrictions on data availability
- For clinical datasets or third party data, please ensure that the statement adheres to our policy

All the experimental data corresponding to compound screenings (dose-response experiments of compounds from literature, HTS, validation of cytotoxicity predictions and validation of generated molecules) and RNA-sequencing are available in the Supplementary Data and at https://github.com/sbnb-irb/cytotoxics_design.git. The data necessary to reproduce all the figures in the study is also included in the GitHub repository. A permanent snapshot of the code and data is archived at Zenodo (https://doi.org/10.5281/zenodo.19655563).

We obtained the publicly available data for this study from several sources. We retrieved cytotoxicity data from GDSC2 (https://www.cancerrxgene.org/). We downloaded the NCI-60 data from CellMiner (https://discover.nci.nih.gov/cellminer/loadDownload.do), 'Compound activity: DTP NCI-60'. We accessed the CTRPv2 dataset via the DepMap portal (https://depmap.org/portal/data_page/?tab=allData#) by selecting 'CTRP CTD^2' from the file set menu. Using the DepMap portal, we also downloaded the 'Proteomics' dataset. We downloaded DrugCell cytotoxicity and mutation data (files 'drug_cell_train.txt', 'drug_cell_test.txt', 'drug_cell_val.txt', and 'cell2mutation.txt') from their GitHub repository (https://github.com/idekerlab/DrugCell/tree/public/data). Finally, we downloaded Bioteque descriptors for all utilized metapaths from the Bioteque web resource (https://bioteque.irbbarcelona.org/downloads).

Research involving human participants, their data, or biological material

Policy information about studies with human participants or human data. See also policy information about sex, gender (identity/presentation), and sexual orientation and race, ethnicity and racism.

Reporting on sex and gender	The study utilized both female (HEK293, U2OS, BxPC-3, and AsPC-1) and male (CAPAN-2, CFPAC-1, PANC-10.05, HPAF-II, and SW1990) cell lines. Sex was not considered a biological variable in the experimental design or analysis, as the study focused on designing new cell line-specific cytotoxics based solely on phenotypic data (previously reported cytotoxicity) and compound bioactivity descriptors, independently of other characteristics of the cell line (i.e., agnostic to genotype or sex). Gender is not applicable to these immortalized cell lines.
Reporting on race, ethnicity, or other socially relevant groupings	Where available, the ethnic/racial origin of the cell lines is documented in the Cellosaurus database (e.g., CFPAC-1 is derived from a Caucasian donor). However, race and ethnicity were not considered as factors in the analysis for the same reasons as stated above.
Population characteristics	No human participants in this study
Recruitment	No recruiting process in this study
Ethics oversight	Not necessary for this study

Note that full information on the approval of the study protocol must also be provided in the manuscript.

Field-specific reporting

Please select the one below that is the best fit for your research. If you are not sure, read the appropriate sections before making your selection.

☒ Life sciences ☐ Behavioural & social sciences ☐ Ecological, evolutionary & environmental sciences

For a reference copy of the document with all sections, see nature.com/documents/nr-reporting-summary-flat.pdf

Life sciences study design

All studies must disclose on these points even when the disclosure is negative.

Sample size	Sample sizes were chosen in accordance to recommendations from the High-throughput platform experts
Data exclusions	No data were excluded
Replication	All the experiments were repeated in technical duplicates or triplicates. Hit molecules were re-tested at least once to ensure reproducibility.
Randomization	compounds and cell lines were placed in different positions in different experiments to avoid any bias
Blinding	experiments were not blinded

Reporting for specific materials, systems and methods

We require information from authors about some types of materials, experimental systems and methods used in many studies. Here, indicate whether each material, system or method listed is relevant to your study. If you are not sure if a list item applies to your research, read the appropriate section before selecting a response.

Materials & experimental systems

n/a	Involved in the study
☒	☐ Antibodies
☐	☒ Eukaryotic cell lines
☒	☐ Palaeontology and archaeology
☒	☐ Animals and other organisms
☒	☐ Clinical data
☒	☐ Dual use research of concern
☒	☐ Plants

Methods

n/a	Involved in the study
☒	☐ ChIP-seq
☒	☐ Flow cytometry
☒	☐ MRI-based neuroimaging

Eukaryotic cell lines

Policy information about cell lines and Sex and Gender in Research

Cell line source(s)	We acquired the pancreatic cancer cell lines BXPC3, CAPAN2, HPAFII, PANC10.05, and SW1990 from the American Type Culture Collection (ATCC), and CFPAC1 and AsPC-1 lines were kindly provided by Dr Cristina Mayor-Ruiz (IRB Barcelona). Additionally, U2OS and HEK293 cell lines were kindly provided by Dr Jens Lüders (IRB Barcelona).
Authentication	None of the cell lines used were authenticated
Mycoplasma contamination	All cell lines tested negative for mycoplasma contamination
Commonly misidentified lines (See ICLAC register)	We did not used any commonly misidentified lines in this project.

Plants

Seed stocks	*Report on the source of all seed stocks or other plant material used. If applicable, state the seed stock centre and catalogue number. If plant specimens were collected from the field, describe the collection location, date and sampling procedures.*
Novel plant genotypes	*Describe the methods by which all novel plant genotypes were produced. This includes those generated by transgenic approaches, gene editing, chemical/radiation-based mutagenesis and hybridization. For transgenic lines, describe the transformation method, the number of independent lines analyzed and the generation upon which experiments were performed. For gene-edited lines, describe the editor used, the endogenous sequence targeted for editing, the targeting guide RNA sequence (if applicable) and how the editor was applied.*
Authentication	*Describe any authentication procedures for each seed stock used or novel genotype generated. Describe any experiments used to assess the effect of a mutation and, where applicable, how potential secondary effects (e.g. second site T-DNA insertions, mosiacism, off-target gene editing) were examined.*
